# Supplementary material for: Evolution of promoter-proximal pausing enabled a new layer of transcription control
Source: bioRxiv. 2024 Oct 12:2023.02.19.529146. Preprint. [Version 2] doi: 10.1101/2023.02.19.529146 (PMC11482795; doi:10.1101/2023.02.19.529146)

bioRxiv preprint doi: https://doi.org/10.1101/2023.02.19.529146; this version posted October 12, 2024. The copyright holder for this preprint (which was not certified by peer review) is the author/funder, who has granted bioRxiv a license to display the preprint in perpetuity. It is made available under aCC-BY 4.0 International license.

Supplemental Materials:

Figure S1: Correlation of experimental replicates.

Pearson (A) and Spearman (B) correlation between biological replicates for ChRO-seq data generated for this project. In some cases biological replicates came from different tissues (D. iulia), sexes (P. marinus), or treatment conditions (D. discoideum, S. purpuratus).

(C) Profiles show the number of PRO-seq reads per species is reported as a function of insert size. A color gradient from orange (depicting highly degraded RNA) to white (depicting lowly degraded RNA) marks the quality of each sample. A degradation ratio score is also reported at the top of each plot. Degradation ratios for *C.elegans* and *A.thaliana* were computed manually using the scripts in Scott *et al.*[83].

Figure S2: Comparability of PRO-seq, GRO-seq, and ChRO-seq.

Box and whiskers plot of pausing indexes in each species. Boxes are clustered by whether any NELF subunits are present (at least one subunit present vs. no subunits present) in each species for which PRO-seq (A, n = 5), GRO-seq (B, n = 3), and ChRO-seq (C, n = 12), were used in this project. A Mann-Whitney test was used to compute p-values.

Figure S3: TSS reannotation.

PRO-seq (blue) and PRO-cap[25] (red) metaprofiles for *S. Pombe*[23] (A), *S. cerevisiae*[23] (B), *D. Melanogaster*[25] (C), and *H. Sapiens*[84] (D). Upper panels use published gene annotations, lower panels use reannotated genes. Note the relative depletion of PRO-cap reads upstream and downstream of the TSS and more focused pause in PRO-seq signal of *D. melanogaster* and *H. sapiens* in re annotated panels.

bioRxiv preprint doi: https://doi.org/10.1101/2023.02.19.529146; this version posted October 12, 2024. The copyright holder for this preprint (which was not certified by peer review) is the author/funder, who has granted bioRxiv a license to display the preprint in perpetuity. It is made available under aCC-BY 4.0 International license.

Figure S4: Clustering species by their pausing index values.

Density maps of pausing indexes for each species. The plots are split into four quantiles and colored accordingly.

Figure S5: Maximum likelihood phylogenies of pausing machinery components.

Red branches: opisthokonts, purple branches: amorpheans (opisthokonts and their sister lineages breviates, apusomonads and amoebozoans), cyan branches: other eukaryotes, black branches: archaea, grey branches: bacteria. For ease of viewing, only taxon names of opisthokonts, and only robust support values (higher than both 95% ultrafast bootstrap and 80% SH-aLRT supports), are shown. Raw phylogenies are provided as supplementary materials [Supplementary Files].

Figure S6: Selected positions of a multiple sequence alignment of NELF-A sequences from selected eukaryotes.

Amino acids are shaded according to hydrophobicity value, where red is most hydrophobic and blue is the most hydrophilic, and numbered according to the Homo sapiens sequence. Locations of the Homo sapiens NELF-A NELFCD-binding domain (black) and NELF-A tentacle (grey) are shown above the sequence. HDAg domains predicted by InterProScan are underlined.

Alignment constructed with MAFFT-L-INS-I, visualized with Geneious® 2023.2.1, and edited with Affinity Designer 2.

Figure S7: Selected positions of a multiple sequence alignment of NELF-E and paralogous sequences from selected opisthokonts.

Paralogous sequences include polyadenylate-binding proteins and nucleolysin TIAR. Amino acids are shaded according to hydrophobicity value, where red is most hydrophobic and blue is the most hydrophilic, and numbered according to the Homo sapiens NELF-E (black numbers) and polyadenylate-binding protein 1 (red) sequences. The RNA Recognition Motif shared by these and other RNA-binding proteins is shown in black above the sequence.

Alignment constructed with MAFFT-L-INS-I, visualized with Geneious® 2023.2.1, and edited with Affinity Designer 2.

Figure S8: Selected positions of a multiple sequence alignment of HEXIM sequences from selected eukaryotes.

Amino acids are shaded according to hydrophobicity value, where red is most hydrophobic and blue is the most hydrophilic, and numbered according to the Homo sapiens HEXIM1 sequence, and trimmed to the Homo sapiens HEXIM1 HEXIM Pfam domain. Arrows show Homo sapiens HEXIM1 residues Pro202, Thr205, Phe207 and Tyr271, crucial for the recruitment and inhibition of P-TEFb in human cells.

Alignment constructed with MAFFT-L-INS-I, visualized with Geneious® 2023.2.1, and edited with Affinity Designer 2.

Figure S9: Association of NELF and HEXIM subunits with pausing index.

Box and whiskers plots show pausing index values in each species. Samples are clustered by the presence or absence of any NELF subunits (A), and both HEXIM subunits (B). A two-sided Mann-Whitney test was used to compute p-values between the PI values.

Figure S10: Pause motif search.

(A) Box and whiskers plots depict enrichment of motif scores for the pause button in each species. Samples are clustered by the presence or absence of NELF-B, -C/D, and -A/E. A two-sided Mann-Whitney test was used to compute p-values.

bioRxiv preprint doi: https://doi.org/10.1101/2023.02.19.529146; this version posted October 12, 2024. The copyright holder for this preprint (which was not certified by peer review) is the author/funder, who has granted bioRxiv a license to display the preprint in perpetuity. It is made available under aCC-BY 4.0 International license.

(B) Metaprofile plot of reads mapping to maternal and paternal alleles for genes with stronger pause motif on the maternal (left) or paternal (right) alleles.
(C) Scatter plot of enrichment motif score of Initiator sequence plotted against the mean pausing index per species (R = 0.105, p = 0.661). Each dot is colored by the number of NELF subunits found in each sample.
(D) Box and whiskers plots depict enrichment of motif scores for the initiator motif in each species. Samples are clustered by the presence or absence of NELF-B, -C/D, and -A/E. A two-sided Mann-Whitney test was used to compute p-values.

Figure S11: *nelfe-FKBP12* homozygous cell line generation.

(A) Schematic of CRISPR design to add the FBBP12 tag at the *nelfe* locus.
(B) PCR validation of CRISPR insertion of the FKBP12 tag.
(C) Microscopy images evaluating the degradation efficiency before (top) and after a 30min treatment with 500nM dTAG-13 (bottom) in the edited and unedited cell lines. Hoechst was used as a nuclear control, while anti-HA antibodies measure the added tag, and anti-NELFE measures the NELF-E protein level. Arrows point out the presence of Feeder cells.
(D) Degradation efficiency of NELF-E as measured by western blotting. b-Actin was used as a loading control, while anti-HA measures the level of NELFE-HA protein. Input denotes the relative amount of total protein loaded.

Figure S12: *nelfb and nelfe-FKBP12* homozygous cell line validation.

(A) Western blot of whole cells following NELF-B (left) or NELF-E (right) degradation with 500nM dTAG-13 for 0 to 24h of treatment.
(B) Western blot validation of chromatin fraction vs nuclear soluble fractionation.
(C) Western blot of NELF-B and -E proteins after degradation of either protein for 1h. Both nuclear-soluble and chromatin-bound proteins were analyzed.
(D) Quantification of western blot signal in (C) for NELF-E after degradation of NELF-B, and vice-versa.

Figure S13: Effect of NELF-B and NELF-E degradation on Pol II distribution.

(A) WashU browser shots at the Nanog gene locus before and after NELF-B and -E degradation.
(B) Heat maps of spike-in normalized PRO-seq signal
(C) Heatmaps of log2 fold changes of normalized PRO-seq signal relative to untreated controls. All heat maps are centered on active TSSs in mESCs.
(D) Metaprofiles of cluster 3 genes at each dTAG time point, the recovery of pause-like behavior between 30 and 60 minutes, and the proto-pause observed in *S. pombe* for reference. The proto-pause region is shaded in the two rightmost panels.

Figure S14: Characterization of transcription recovery clusters after NELF-B degradation.

(A) Bar plots depict the percentage of transcribed enhancers and gene promoters in each cluster defined in Figure 3E.
(B) Enrichment profiles of the pause motif published in Watts *et al*., Am J Hum Genet (2019) plotted in a 1kb window centered on TSSs found in each of the clusters defined in Figure 3E.
(C) Violin plots depict log10 transformed initiation (right) or pause release (left) rates in each cluster. A two-sided Mann-Whitney test was used to compute p-values, where n.s. defines non-significant p-values, and (***) p-values < 2.2e-16.
(D) Plots depict the enrichment of the TATA box, Initiator, MTE, and DPE sequence motifs in each cluster in Figure 3E.

bioRxiv preprint doi: https://doi.org/10.1101/2023.02.19.529146; this version posted October 12, 2024. The copyright holder for this preprint (which was not certified by peer review) is the author/funder, who has granted bioRxiv a license to display the preprint in perpetuity. It is made available under aCC-BY 4.0 International license.

(E) Meta profiles depict the enrichment of TBP, TAF-12, TFIIA, TFIIB, H3K9ac, and Med1 per cluster.

Figure S15: Pol II trickles into gene bodies effect after NELF-B degradation.

Heatmaps of log2 fold changes in PRO-seq signal in NELF-B tagged cell lines at all TSSs, Clusters 1, 2, and 3 (in this order from top to bottom rows). The heatmaps depict log2 fold change relative for the following comparisons (from left to right columns): untreated PRO-seq signal, log2 fold change for 30min/0min, 60min/0min, and 60min/30min of dTAG-13 treatment.

Figure S16: Correlations between heat shock PRO-seq data.

(A) Principal component analysis (PCA) of non-heat shock (NHS), dTAG-13 treatment (dTAG), heat shock (HS), and a pre-treatment of dTAG-13 followed by heat shock (HS+dTAG).

(B) Clustered dendrogram of spearman correlations (rho) between the NELF-B and NELF-E HS and NHS conditions before any protein degradation.

(C) Clustered dendrogram of Pearson correlations (r) between all three independent replicates of HS and NHS in both NELF-B and NELF-E cell lines before degradation. R1, r2, and r3 represent replicate numbers.

Figure S17: Studying the heat shock response after NELF-B or NELF-E degradation.

WashU browser shots at the heat-triggered genes (Hist1h3b, Hsp1h1, Hist1h3b) in the NELF-B (A) and NELF-E (B) edited cell lines.

Figure S18: Assessing the heat shock response in *nelfb-fkbp12* and *nelfe-fkbp12* homozygous cell lines.

(A) & (C) MA plots show the log2 fold change in gene body PRO-seq signal when comparing dTAG-13 treatment (dTAG) with non-heat shock (NHS), heat shock (HS) with NHS, and the dTAG-13 pre-treatment followed by HS with NHS in the *nelfb-fkbp12* (A) and the *nelfb-fkbp12* (C) cell lines.

(B) & (D) Bar plots depict the percentage of upregulated (red), downregulated (blue), and unchanged (gray) genes in the *nelfb-fkbp12* (B) and the *nelfb-fkbp12* (D) cell lines.

(E) Violin plots show the log2 fold change in gene body PRO-seq signal when comparing the pre-treatment degradation of either NELF-B (left) or NELF-E (right) at genes known to be upregulated (blue) or downregulated (red) after regular heat stress.

(F) Heatmaps of log2 fold changes in PRO-seq signal in NELF-B (left) and NELF-E tagged (right) cell lines. The heatmaps rows depict fold changes relative to NHS for the following treatments: dTAG-13 treatment alone, HS alone, and dual treatment of dTAG-13 and HS.

(G-H) Bar graphs at the heat-shock dependent genes that show a defect in up-regulation after NELF depletion. The graphs show the frequency of log2 fold changes in PRO-seq data when comparing the dual treatment of dTAG-13 followed by heat shock with a non-heat shock control. Data is presented for both the *nelfb-FKBP12* (left) and the *nelfe-FKBP12* (right) cell lines.

bioRxiv preprint doi: https://doi.org/10.1101/2023.02.19.529146; this version posted October 12, 2024. The copyright holder for this preprint (which was not certified by peer review) is the author/funder, who has granted bioRxiv a license to display the preprint in perpetuity. It is made available under aCC-BY 4.0 International license.

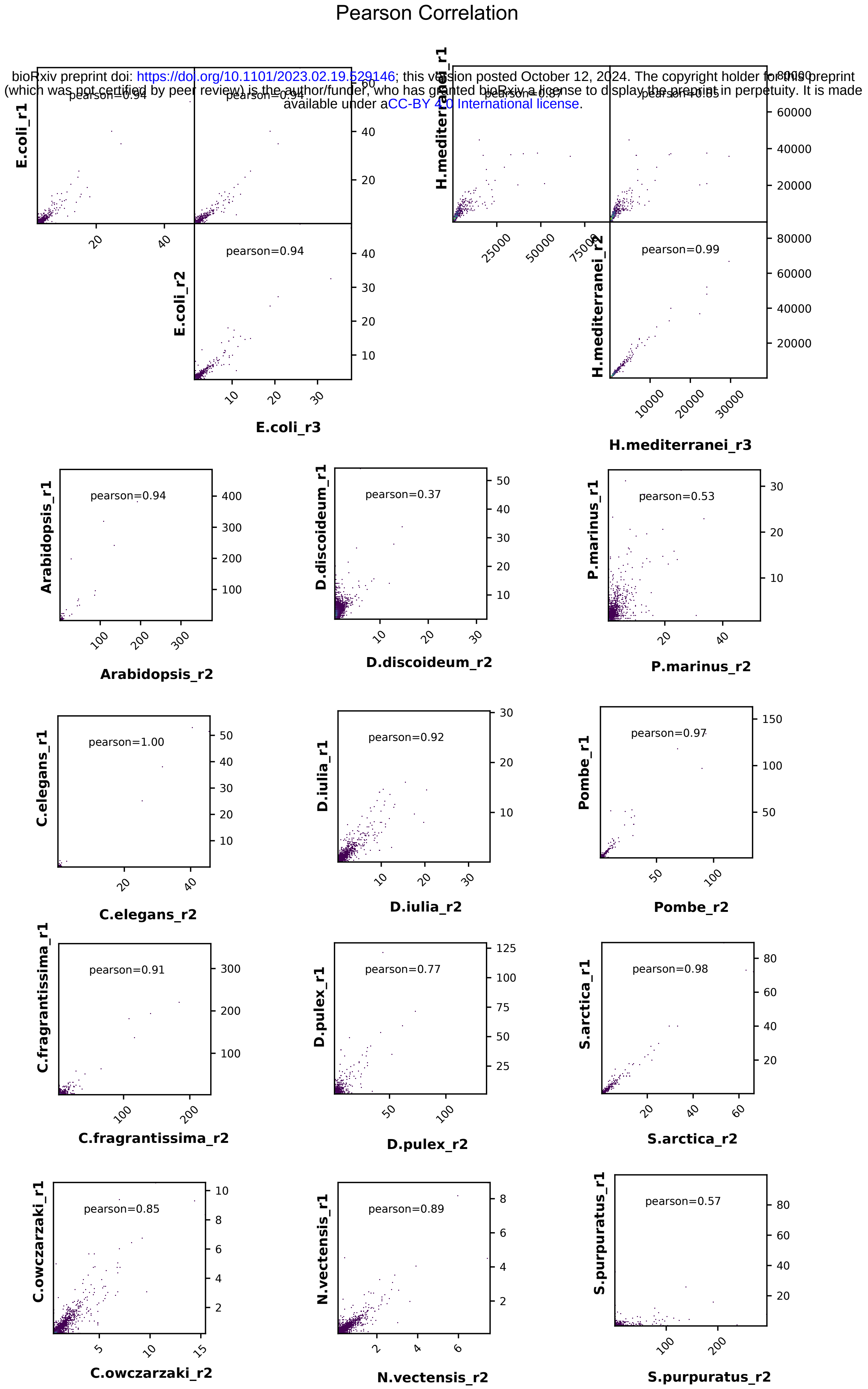

bioRxiv preprint doi: https://doi.org/10.1101/2023.02.19.529146; this version posted October 12, 2024. The copyright holder for this preprint (which was not certified by peer review) is the author/funder, who has granted bioRxiv a license to display the preprint in perpetuity. It is made available under aCC-BY 4.0 International license.

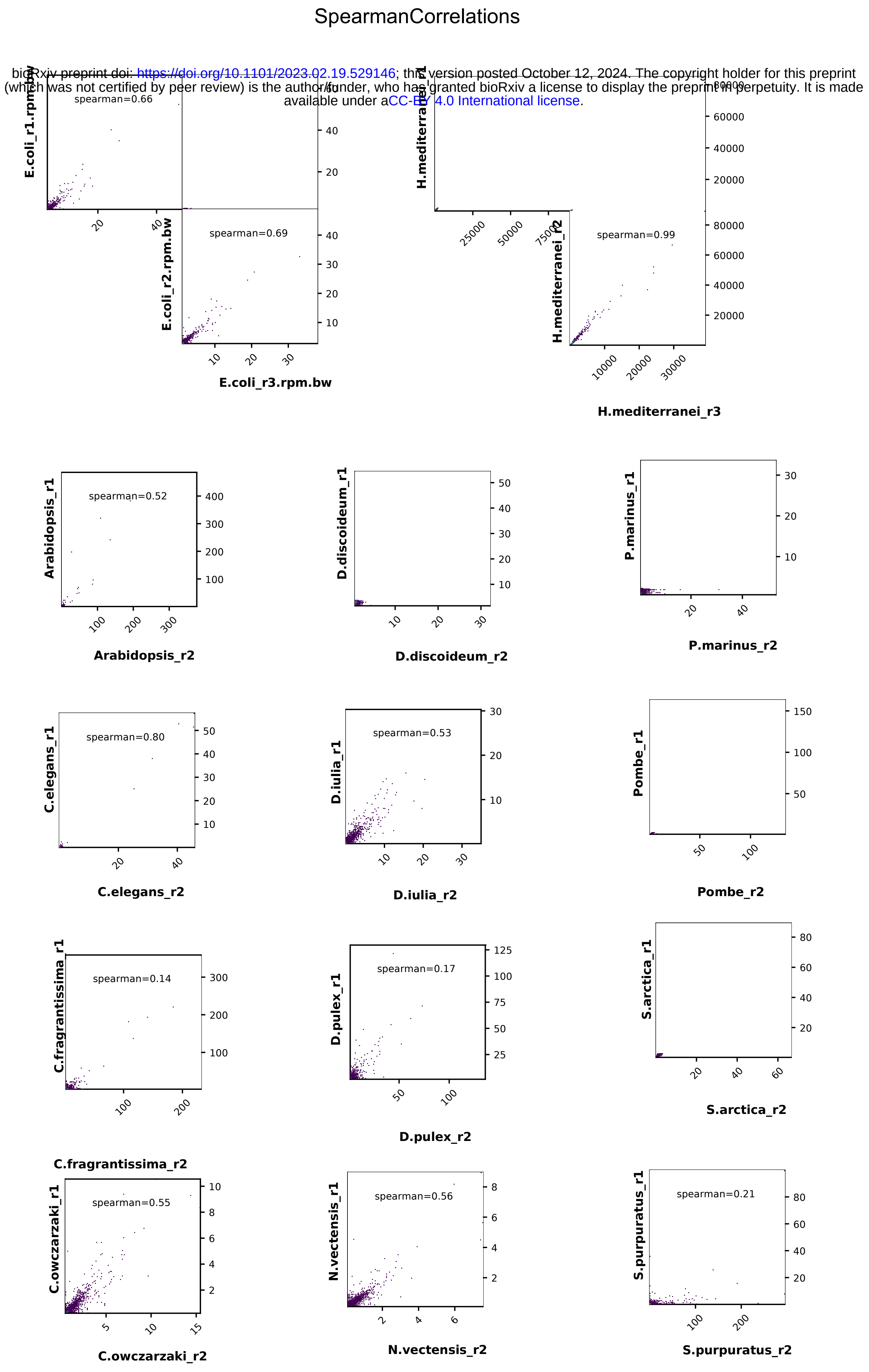

bioRxiv preprint doi: https://doi.org/10.1101/2023.02.19.529146; this version posted October 12, 2024. The copyright holder for this preprint (which was not certified by peer review) is the author/funder, who has granted bioRxiv a license to display the preprint in perpetuity. It is made available under aCC-BY 4.0 International license.

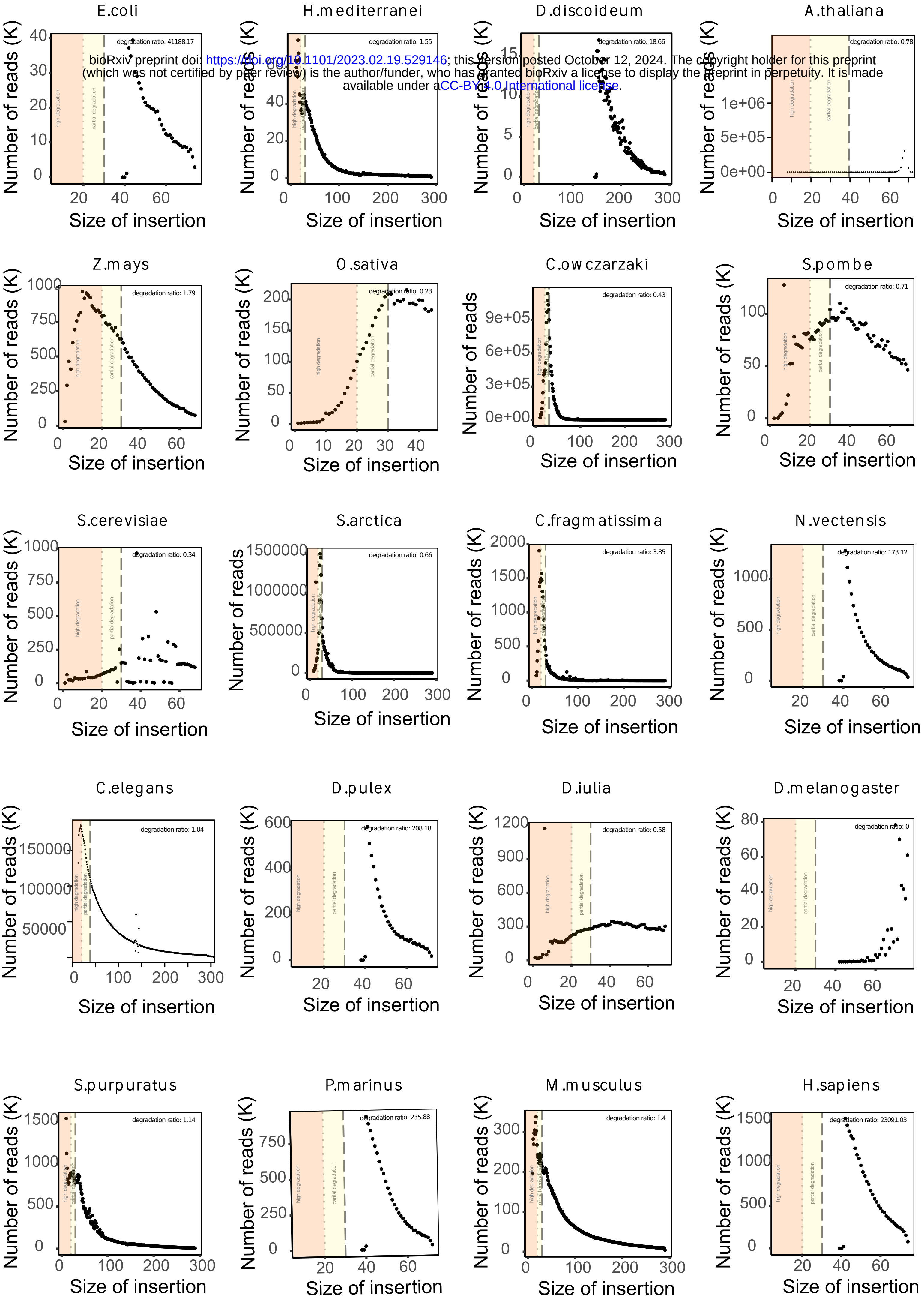

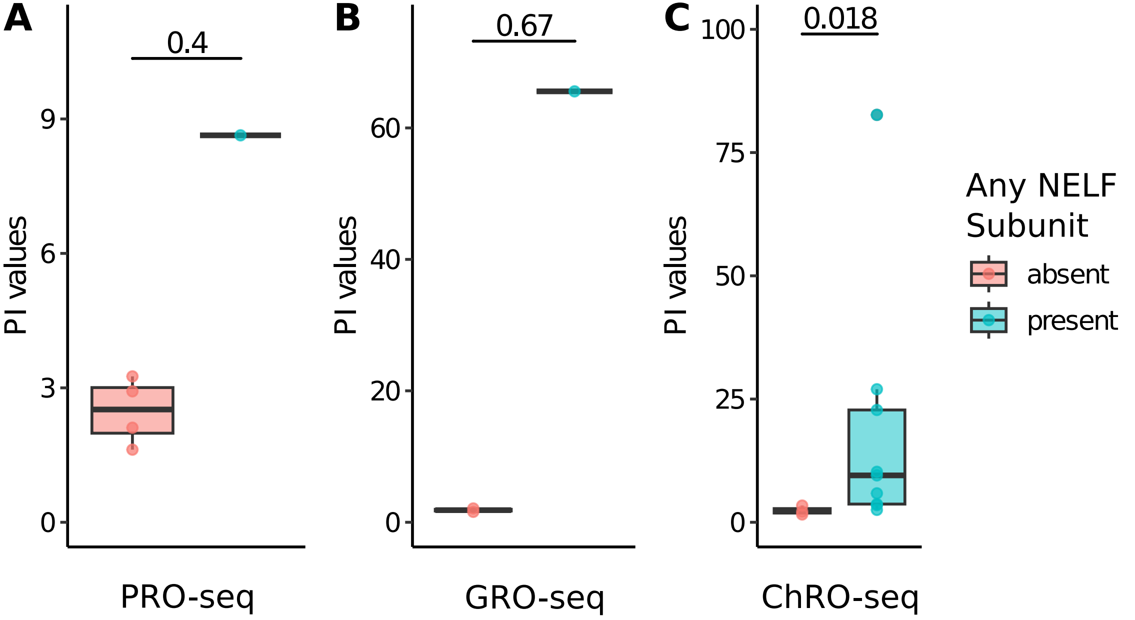

A
0.4
9
6
3
0
PI values
PRO-seq
B
0.67
60
40
20
0
PI values
GRO-seq
C
0.018
100
75
50
25
0
PI values
ChRO-seq
Any NELF Subunit
absent
present

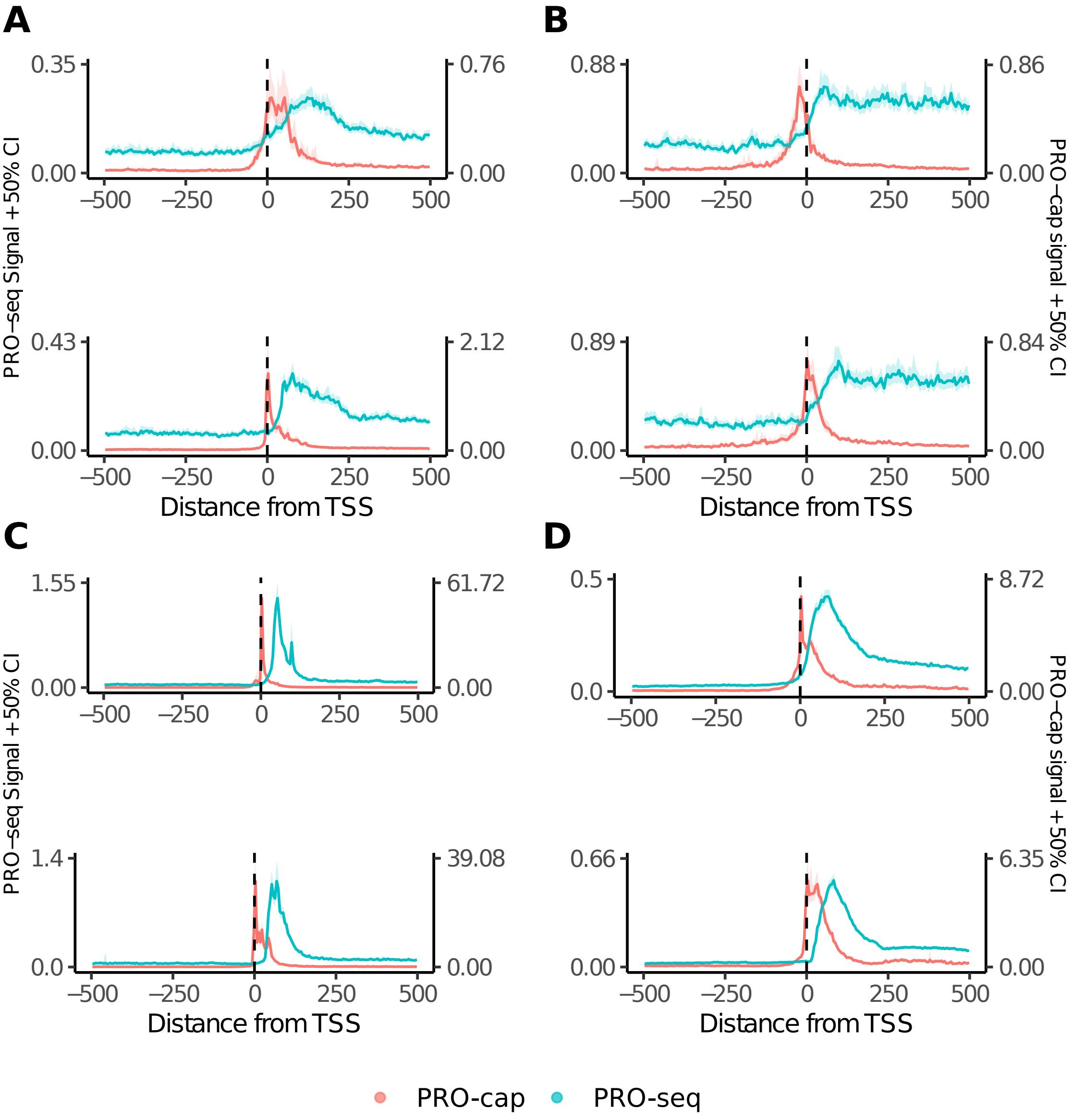
A
B
C
D
0.35
0.00
0.76
0.00
0.88
0.00
0.86
0.00
0.43
0.00
2.12
0.00
0.89
0.00
0.84
0.00
1.55
0.00
61.72
0.00
0.5
0.0
8.72
0.00
1.4
0.0
39.08
0.00
0.66
0.00
6.35
0.00
–500
–250
0
250
500
Distance from TSS
PRO–seq Signal +50% CI
PRO–cap signal +50% CI
PRO-cap
PRO-seq

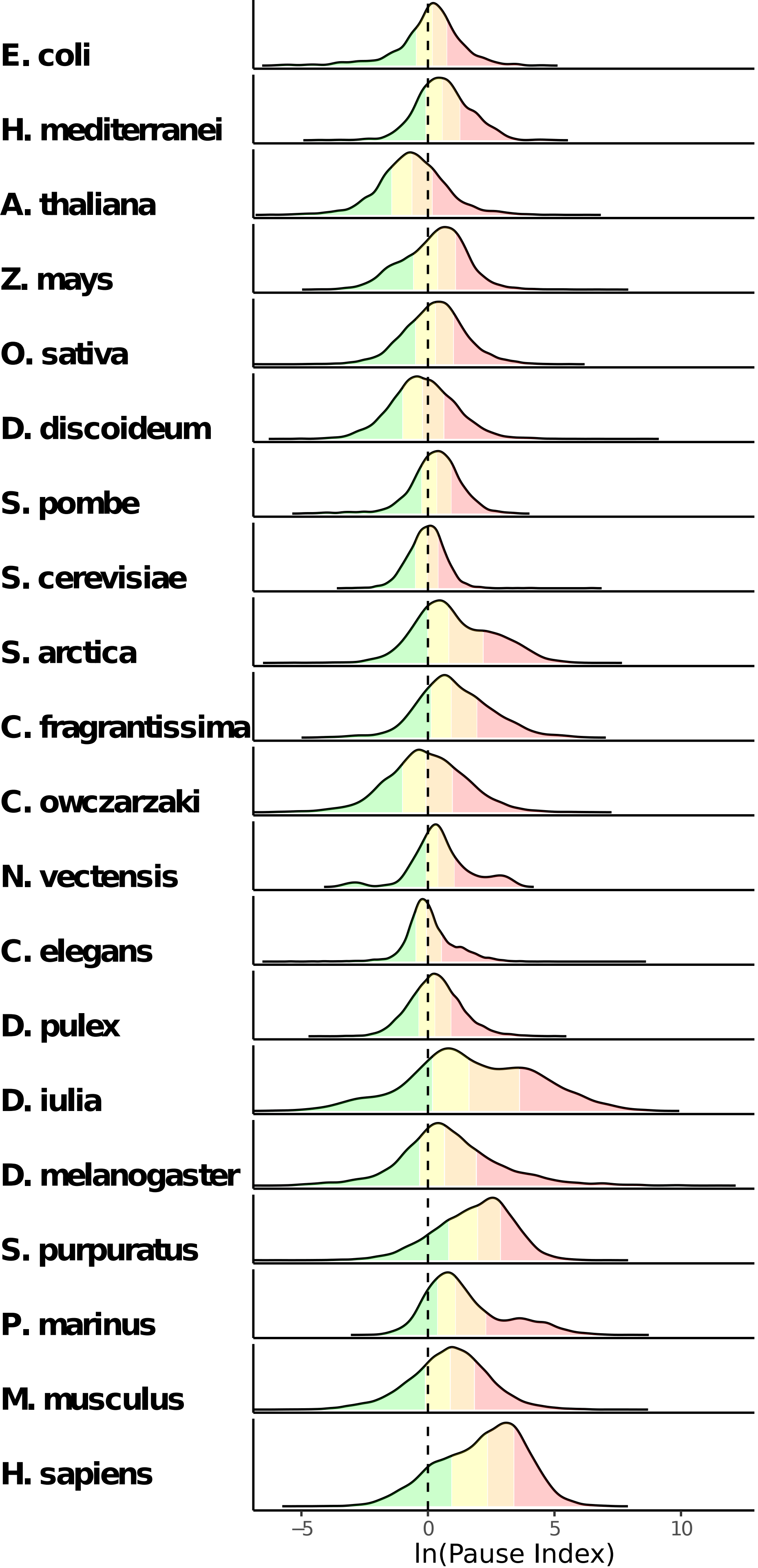
E. coli
H. mediterranei
A. thaliana
Z. mays
O. sativa
D. discoideum
S. pombe
S. cerevisiae
S. arctica
C. fragrantissima
C. owczarzaki
N. vectensis
C. elegans
D. pulex
D. iulia
D. melanogaster
S. purpuratus
P. marinus
M. musculus
H. sapiens
−5
0
5
10
ln(Pause Index)

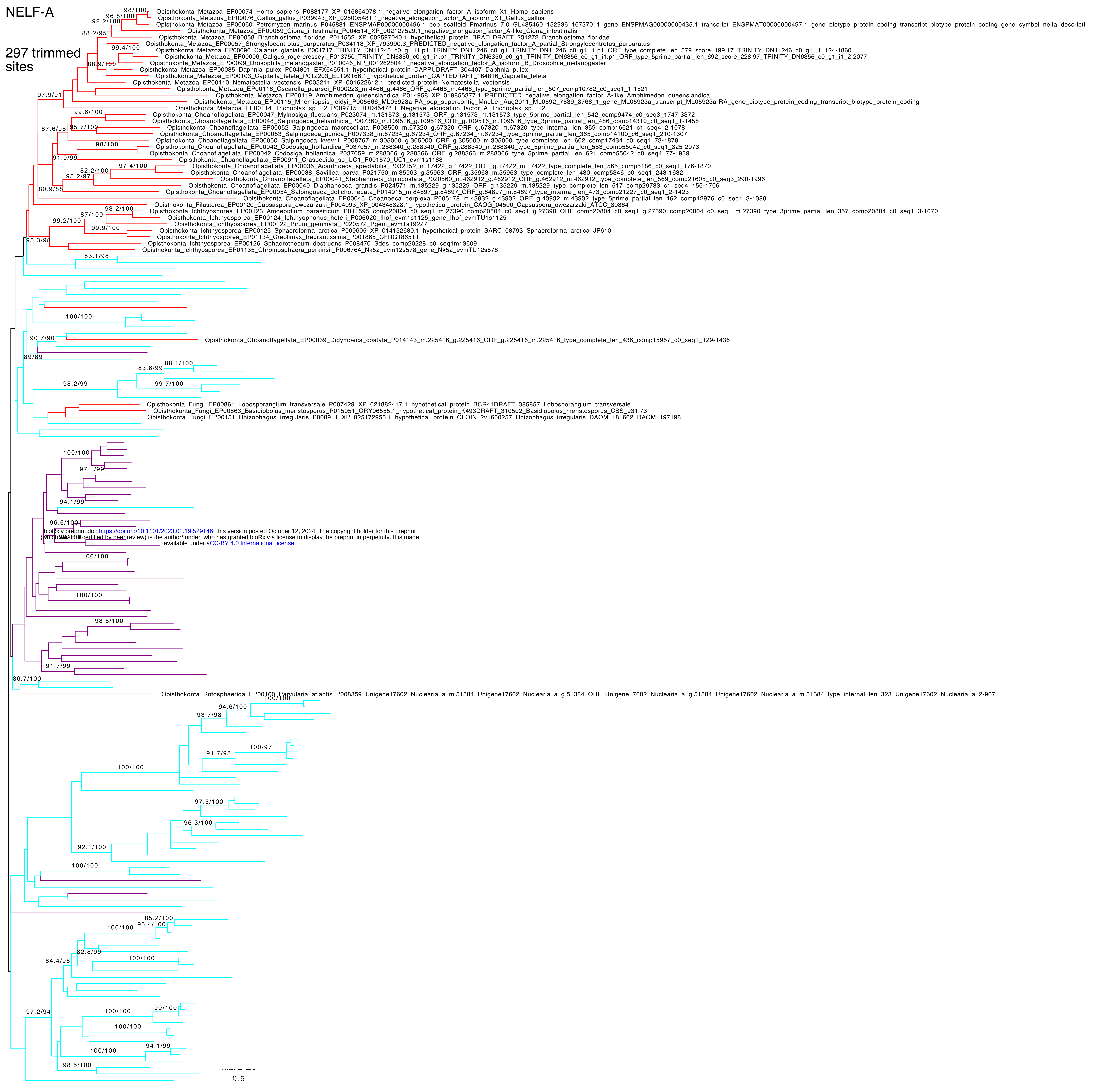

bioRxiv preprint doi: https://doi.org/10.1101/2023.02.19.529146; this version posted October 12, 2024. The copyright holder for this preprint (which was not certified by peer review) is the author/funder, who has granted bioRxiv a license to display the preprint in perpetuity. It is made available under aCC-BY 4.0 International license.

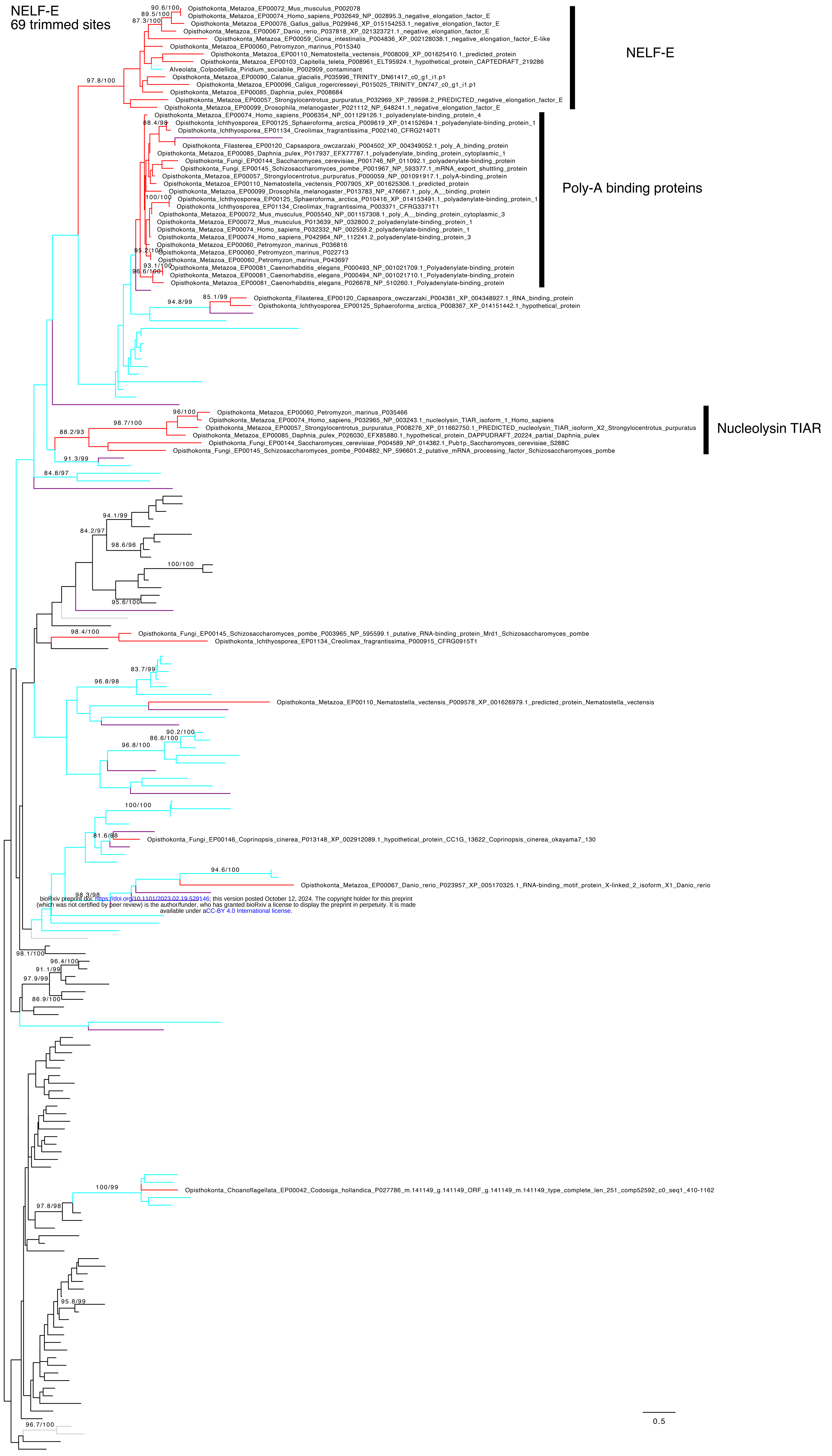

bioRxiv preprint doi: https://doi.org/10.1101/2023.02.19.529146; this version posted October 12, 2024. The copyright holder for this preprint (which was not certified by peer review) is the author/funder, who has granted bioRxiv a license to display the preprint in perpetuity. It is made available under aCC-BY 4.0 International license.

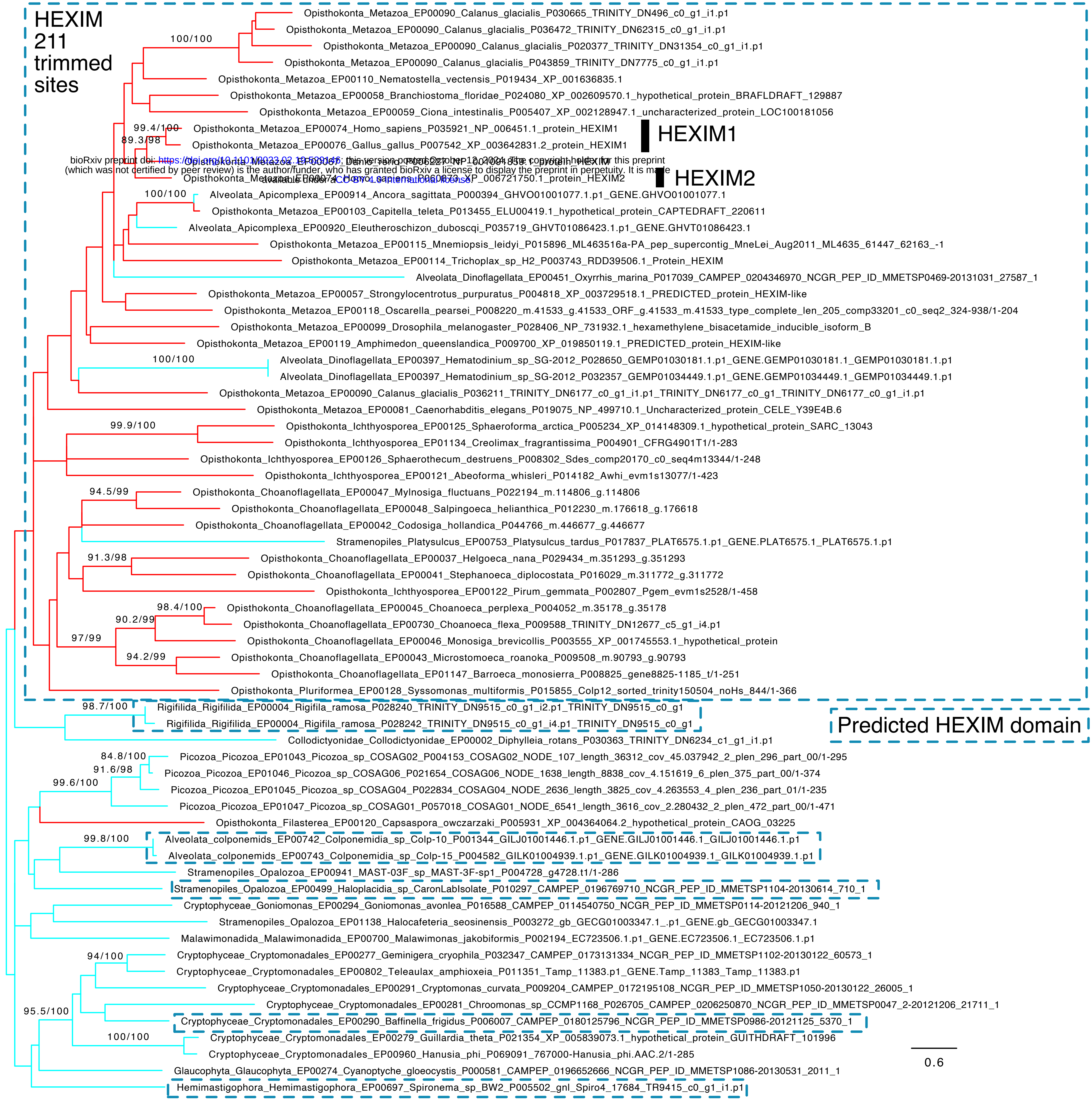

bioRxiv preprint doi: https://doi.org/10.1101/2023.02.19.529146; this version posted October 12, 2024. The copyright holder for this preprint (which was not certified by peer review) is the author/funder, who has granted bioRxiv a license to display the preprint in perpetuity. It is made available under aCC-BY 4.0 International license.

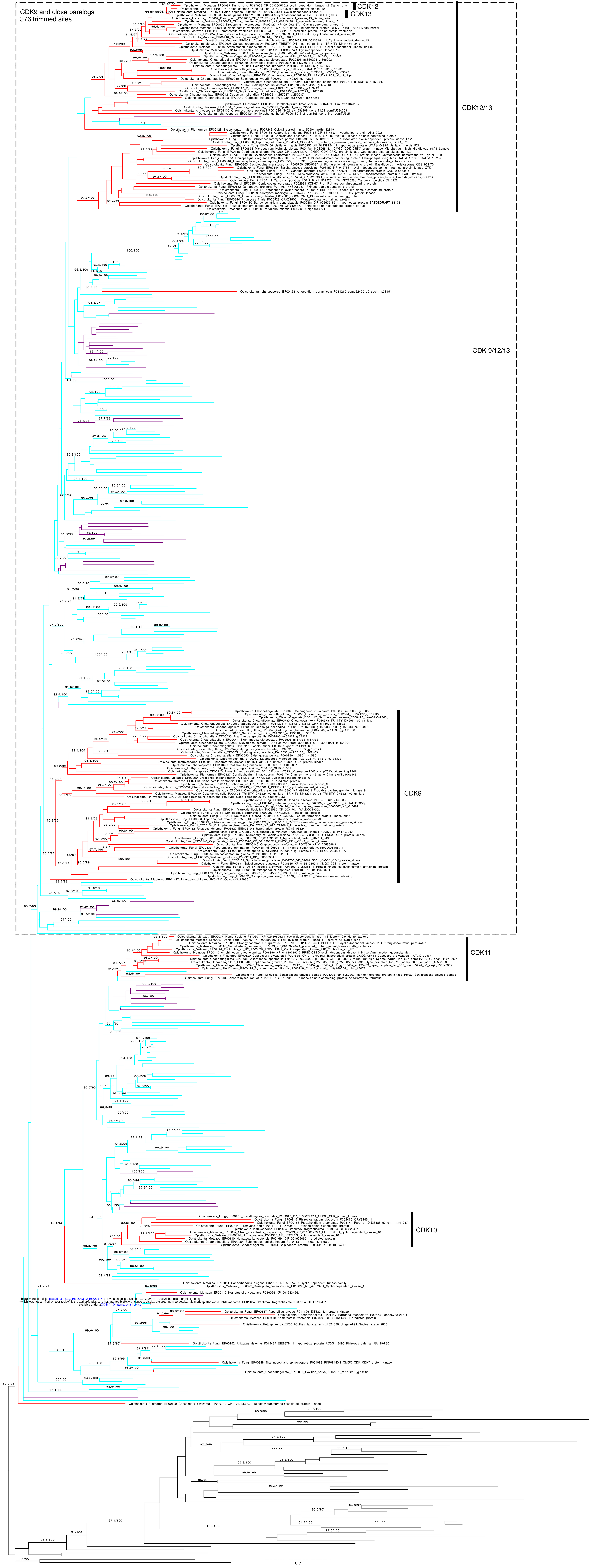

bioRxiv preprint doi: https://doi.org/10.1101/2023.02.10.529146; this version posted October 12, 2024. The copyright holder for this preprint (which was not certified by peer review) is the author/funder, who has granted bioRxiv a license to display the preprint in perpetuity. It is made available under aCC-BY 4.0 International license.

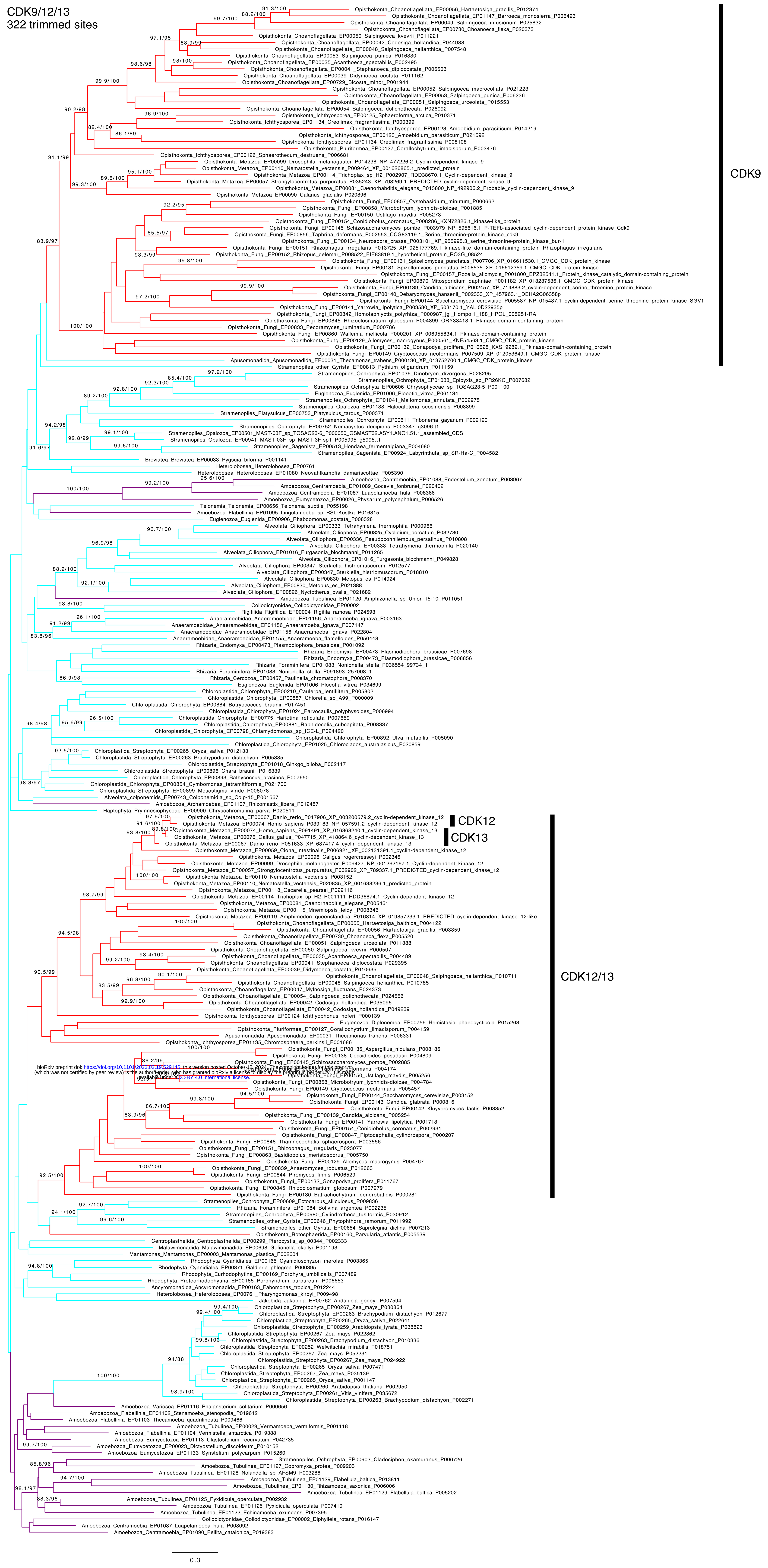

bioRxiv preprint doi: https://doi.org/10.1101/2023.02.19.529146; this version posted October 12, 2023. The copyright holder for this preprint (which was not certified by peer review) is the author/funder, who has granted bioRxiv a license to display the preprint in perpetuity. It is made available under aCC-BY 4.0 International license.

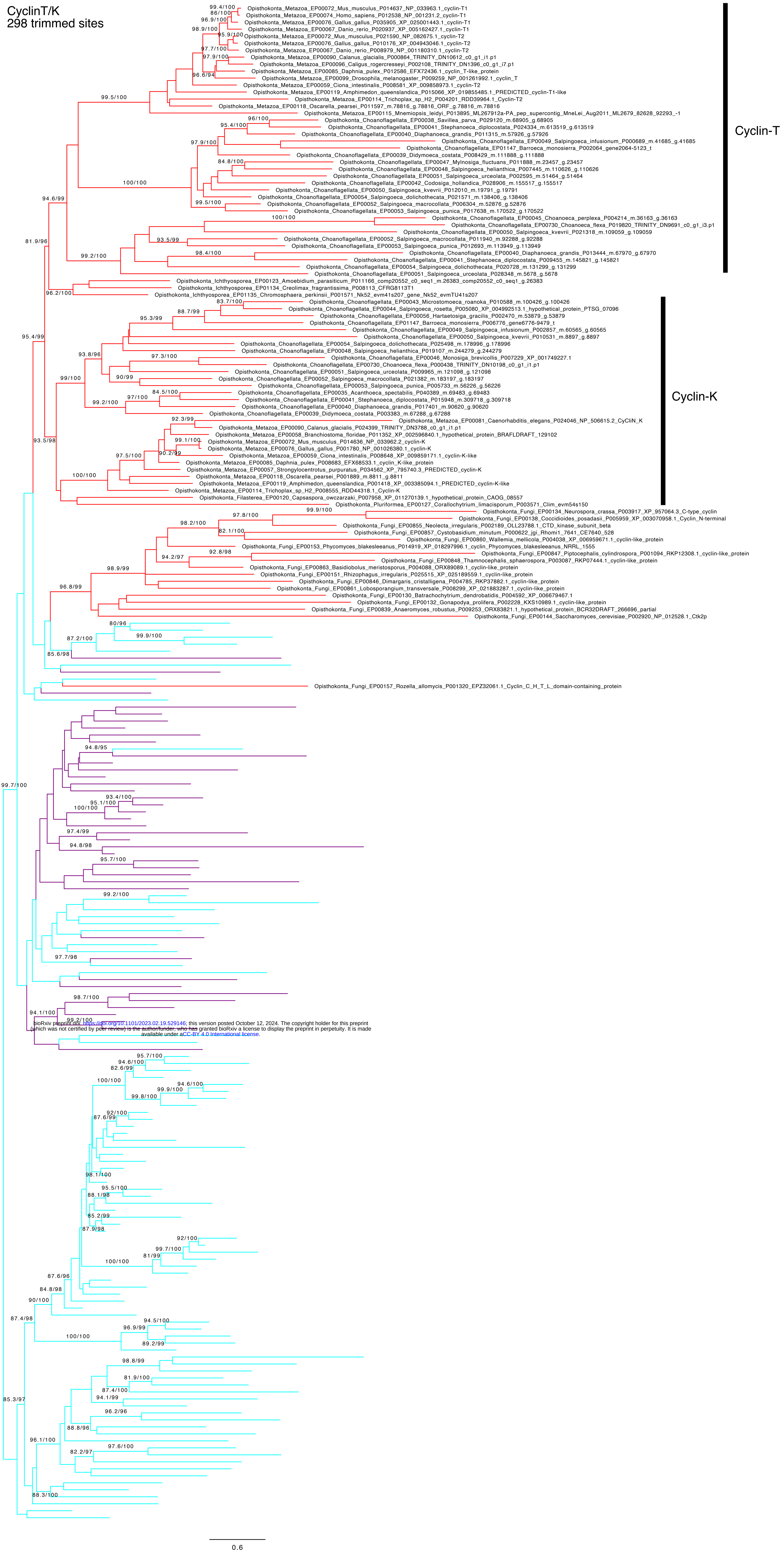

bioRxiv preprint doi: https://doi.org/10.1101/2023.02.19.529146; this version posted October 12, 2024. The copyright holder for this preprint (which was not certified by peer review) is the author/funder, who has granted bioRxiv a license to display the preprint in perpetuity. It is made available under aCC-BY 4.0 International license.

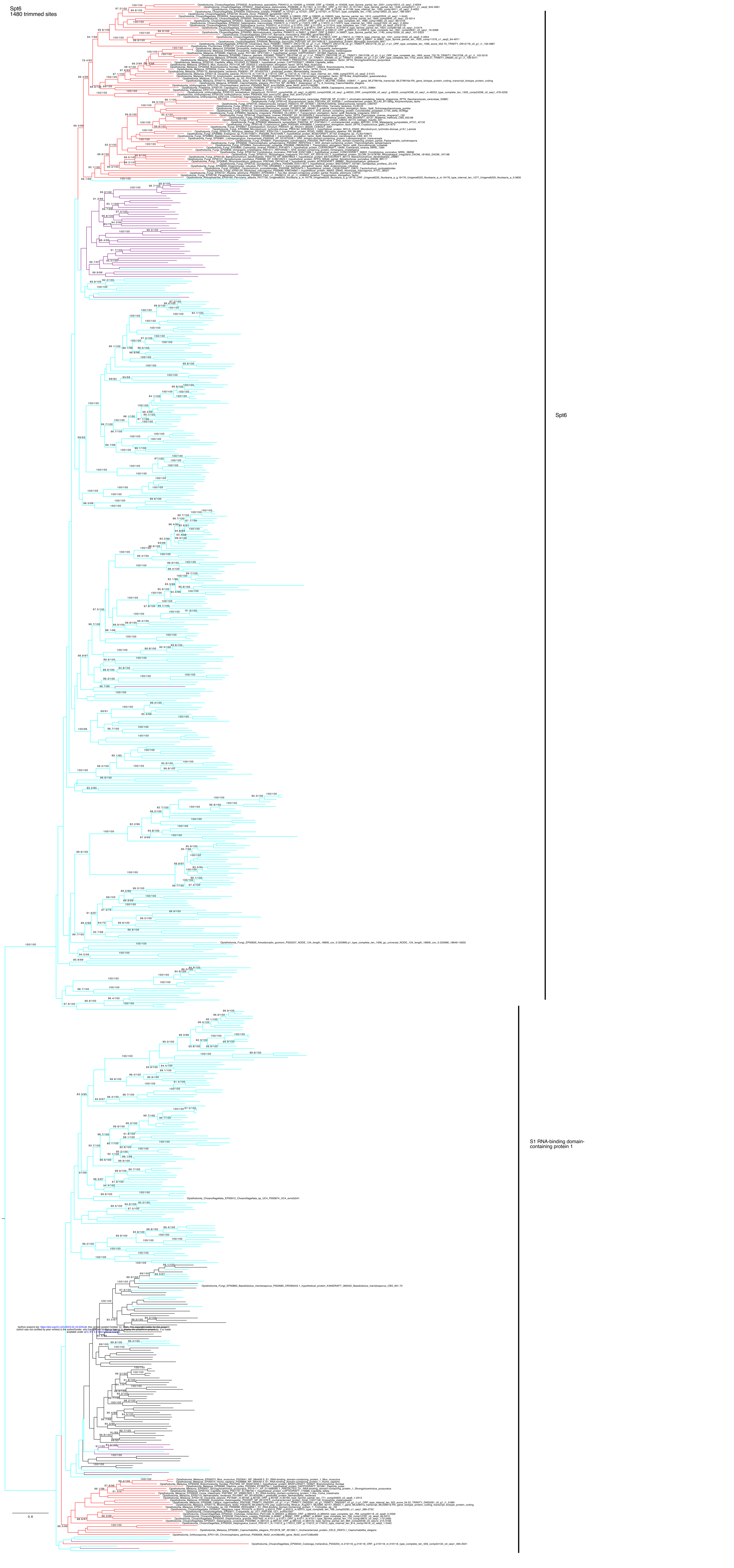

bioRxiv preprint doi: https://doi.org/10.1101/2023.03.19.529246; this version posted October 12, 2023. The copyright holder for this preprint (which was not certified by peer review) is the author/funder, who has granted bioRxiv a license to display the preprint in perpetuity. It is made available under aCC-BY 4.0 International license.

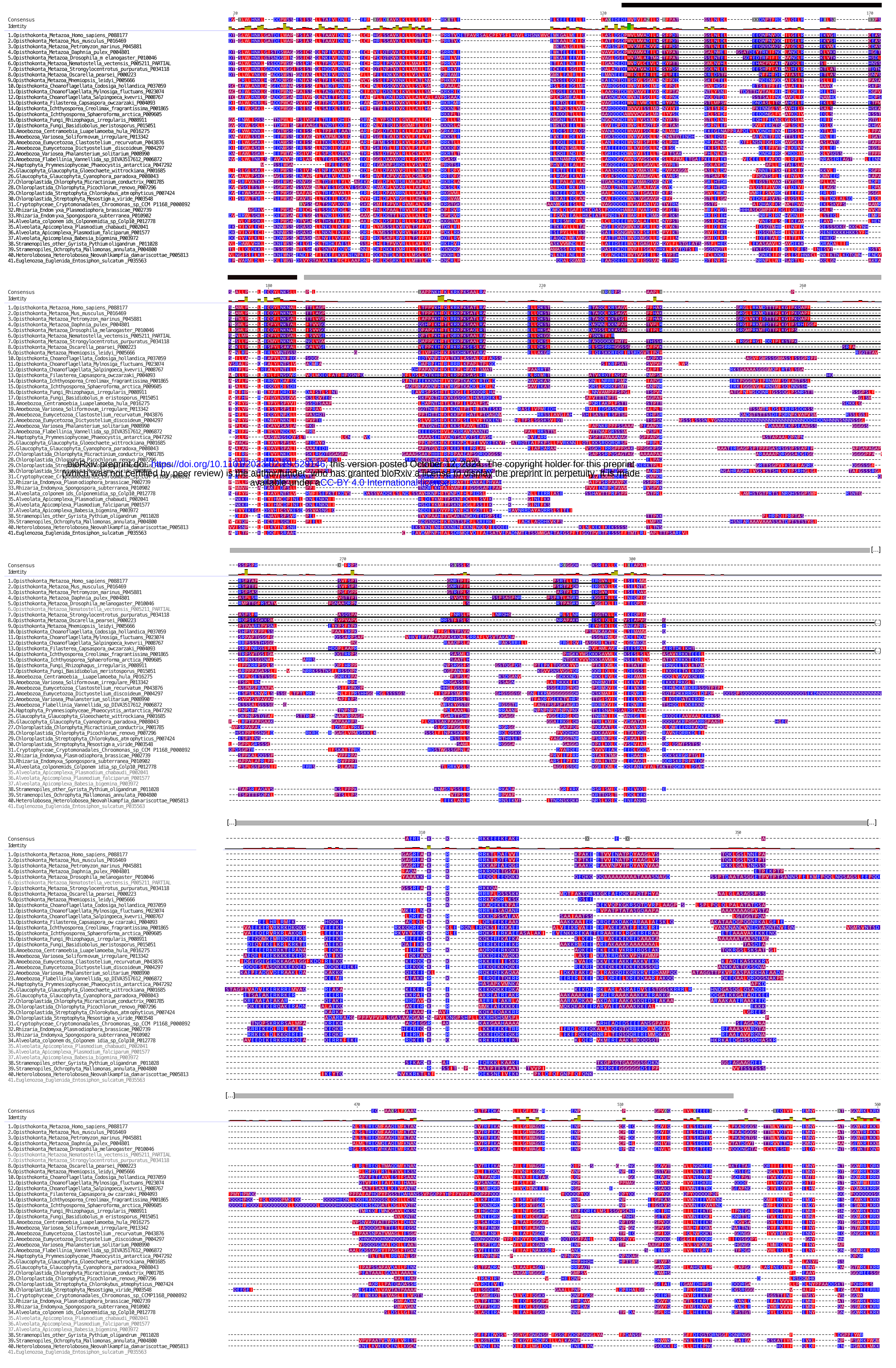

bioRxiv preprint doi: https://doi.org/10.11[illegible]; this version posted October [illegible]. The copyright holder for this preprint (which was not certified by peer review) is the author/funder, who has granted bioRxiv a license to display the preprint in perpetuity. It is made available under aCC-BY 4.0 International license.

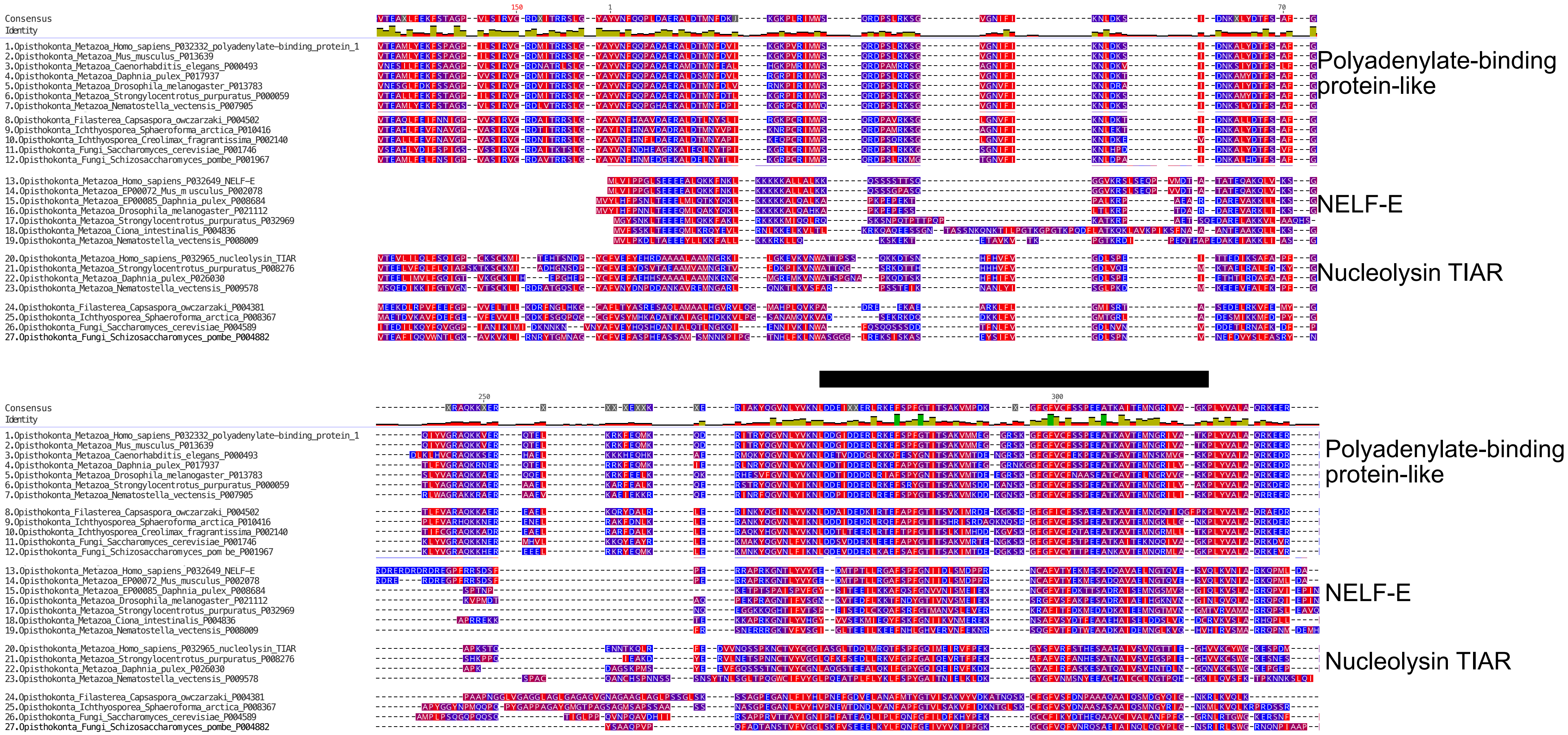
Consensus
Identity
1.Opisthokonta_Metazoa_Homo_sapiens_P032332_polyadenylate-binding_protein_1
2.Opisthokonta_Metazoa_Mus_musculus_P013639
3.Opisthokonta_Metazoa_Caenorhabditis_elegans_P000493
4.Opisthokonta_Metazoa_Daphnia_pulex_P017937
5.Opisthokonta_Metazoa_Drosophila_melanogaster_P013783
6.Opisthokonta_Metazoa_Strongylocentrotus_purpuratus_P000059
7.Opisthokonta_Metazoa_Nematostella_vectensis_P007905
8.Opisthokonta_Filasterea_Capsaspora_owczarzaki_P004502
9.Opisthokonta_Ichthyosporea_Sphaeroforma_arctica_P010416
10.Opisthokonta_Ichthyosporea_Creolimax_fragrantissima_P002140
11.Opisthokonta_Fungi_Saccharomyces_cerevisiae_P001746
12.Opisthokonta_Fungi_Schizosaccharomyces_pombe_P001967
13.Opisthokonta_Metazoa_Homo_sapiens_P032649_NELF-E
14.Opisthokonta_Metazoa_EP00072_Mus_musculus_P002078
15.Opisthokonta_Metazoa_EP00085_Daphnia_pulex_P008684
16.Opisthokonta_Metazoa_Drosophila_melanogaster_P021112
17.Opisthokonta_Metazoa_Strongylocentrotus_purpuratus_P032969
18.Opisthokonta_Metazoa_Ciona_intestinalis_P004836
19.Opisthokonta_Metazoa_Nematostella_vectensis_P008009
20.Opisthokonta_Metazoa_Homo_sapiens_P032965_nucleolysin_TIAR
21.Opisthokonta_Metazoa_Strongylocentrotus_purpuratus_P008276
22.Opisthokonta_Metazoa_Daphnia_pulex_P026030
23.Opisthokonta_Metazoa_Nematostella_vectensis_P009578
24.Opisthokonta_Filasterea_Capsaspora_owczarzaki_P004381
25.Opisthokonta_Ichthyosporea_Sphaeroforma_arctica_P008367
26.Opisthokonta_Fungi_Saccharomyces_cerevisiae_P004589
27.Opisthokonta_Fungi_Schizosaccharomyces_pombe_P004882
Polyadenylate-binding protein-like
NELF-E
Nucleolysin TIAR
150
1
70
250
300

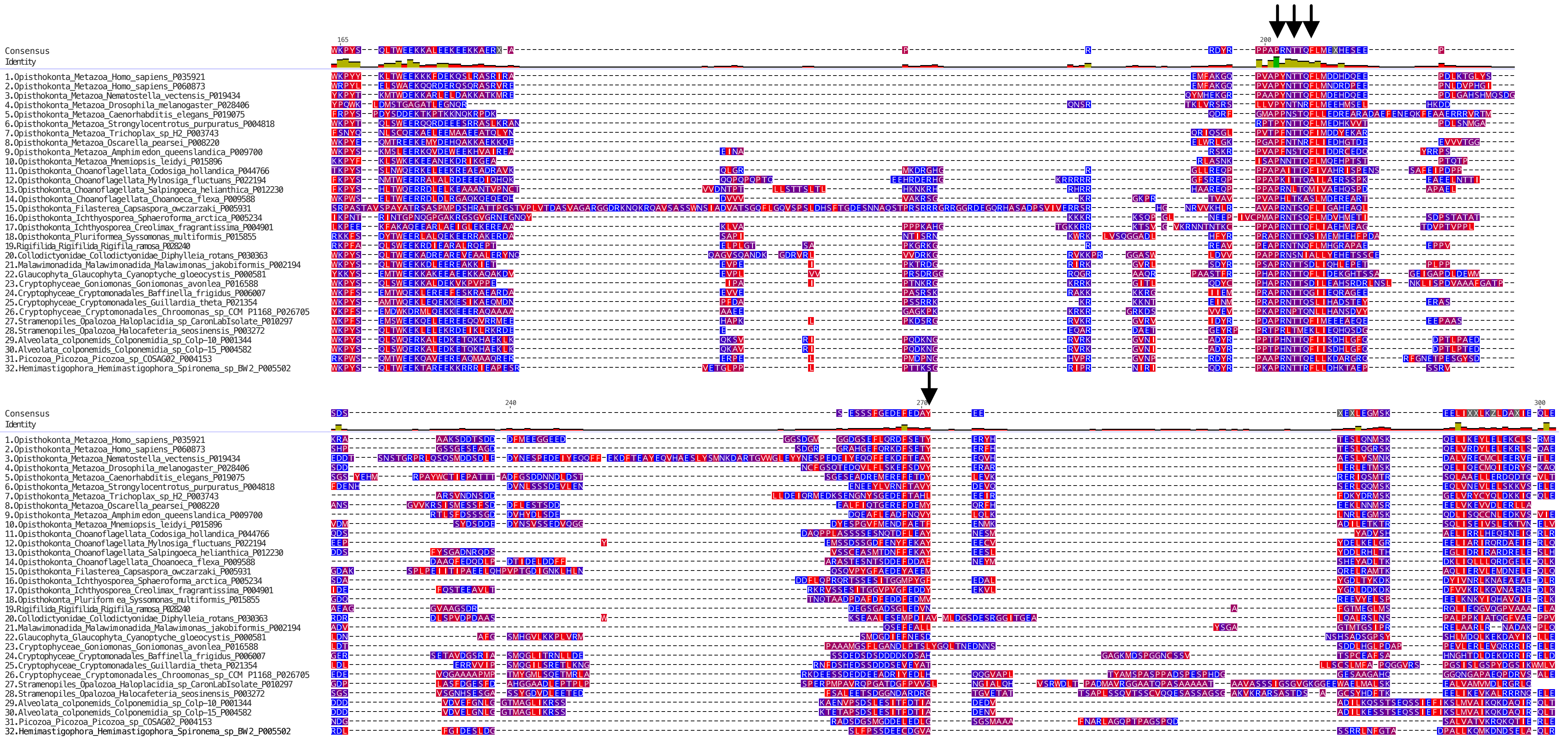

165
200
Consensus
Identity
1.Opisthokonta_Metazoa_Homo_sapiens_P035921
2.Opisthokonta_Metazoa_Homo_sapiens_P060873
3.Opisthokonta_Metazoa_Nematostella_vectensis_P019434
4.Opisthokonta_Metazoa_Drosophila_melanogaster_P028406
5.Opisthokonta_Metazoa_Caenorhabditis_elegans_P019075
6.Opisthokonta_Metazoa_Strongylocentrotus_purpuratus_P004818
7.Opisthokonta_Metazoa_Trichoplax_sp_H2_P003743
8.Opisthokonta_Metazoa_Oscarella_pearsei_P008220
9.Opisthokonta_Metazoa_Amphimedon_queenslandica_P009700
10.Opisthokonta_Metazoa_Mnemiopsis_leidyi_P015896
11.Opisthokonta_Choanoflagellata_Codosiga_hollandica_P044766
12.Opisthokonta_Choanoflagellata_Mylnosiga_fluctuans_P022194
13.Opisthokonta_Choanoflagellata_Salpingoeca_helianthica_P012230
14.Opisthokonta_Choanoflagellata_Choanoeca_flexa_P009588
15.Opisthokonta_Filasterea_Capsaspora_owczarzaki_P005931
16.Opisthokonta_Ichthyosporea_Sphaeroforma_arctica_P005234
17.Opisthokonta_Ichthyosporea_Creolimax_fragrantissima_P004901
18.Opisthokonta_Pluriformea_Syssomonas_multiformis_P015855
19.Rigifilida_Rigifilida_Rigifila_ramosa_P028240
20.Collodictyonidae_Collodictyonidae_Diphylleia_rotans_P030363
21.Malawimonadida_Malawimonadida_Malawimonas_jakobiformis_P002194
22.Glaucophyta_Glaucophyta_Cyanoptyche_gloeocystis_P000581
23.Cryptophyceae_Goniomonas_Goniomonas_avonlea_P016588
24.Cryptophyceae_Cryptomonadales_Baffinella_frigidus_P006007
25.Cryptophyceae_Cryptomonadales_Guillardia_theta_P021354
26.Cryptophyceae_Cryptomonadales_Chroomonas_sp_CCM P1168_P026705
27.Stramenopiles_Opalozoa_Haloplacidia_sp_CaronLabIsolate_P010297
28.Stramenopiles_Opalozoa_Halocafeteria_seosinensis_P003272
29.Alveolata_colponemids_Colponemidia_sp_Colp-10_P001344
30.Alveolata_colponemids_Colponemidia_sp_Colp-15_P004582
31.Picozoa_Picozoa_Picozoa_sp_COSAG02_P004153
32.Hemimastigophora_Hemimastigophora_Spironema_sp_BW2_P005502
240
270
300
Consensus
Identity

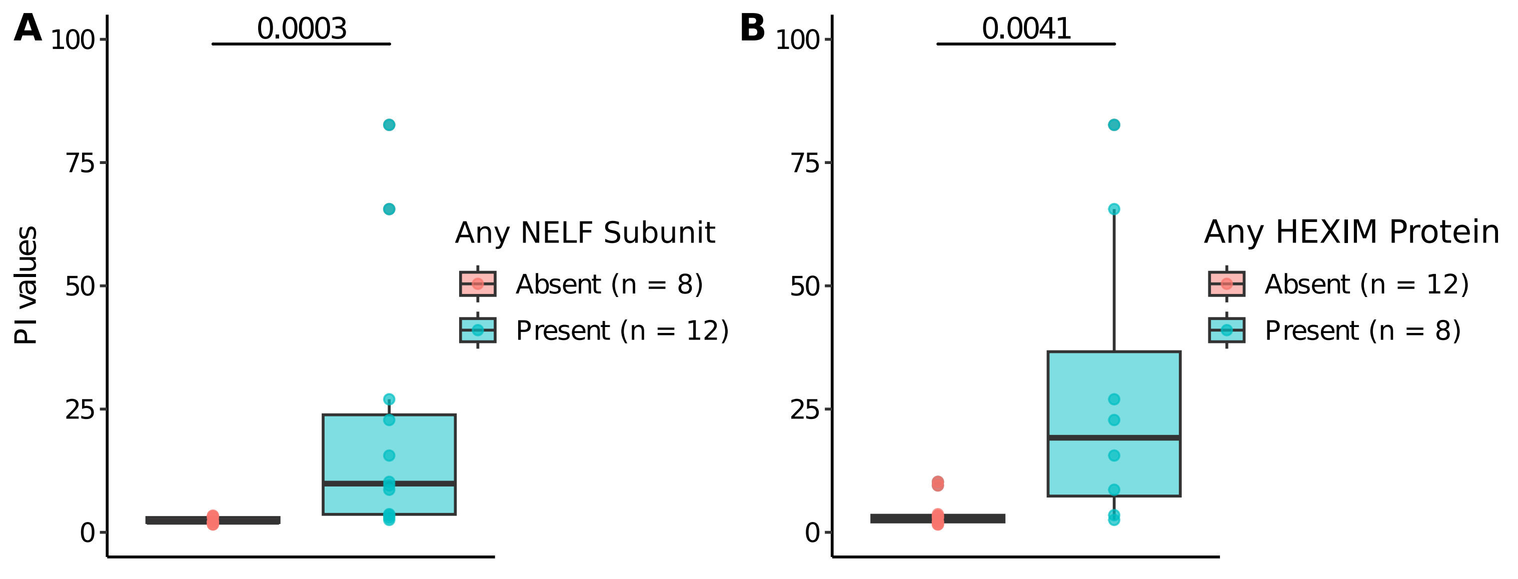

A
B
0.0003
0.0041
100
75
50
25
0
PI values
Any NELF Subunit
Absent (n = 8)
Present (n = 12)
Any HEXIM Protein
Absent (n = 12)
Present (n = 8)

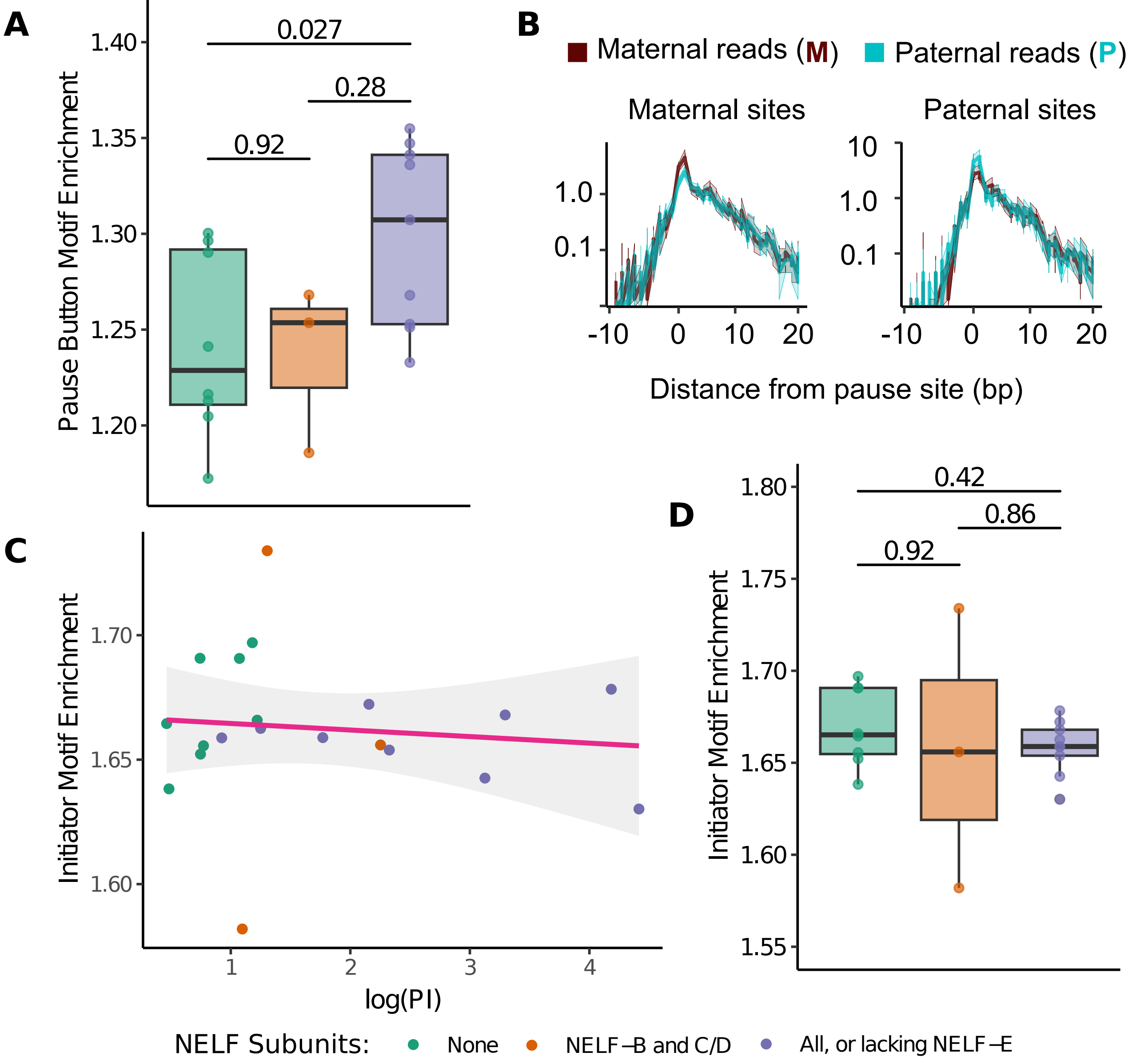
A
0.027
0.28
0.92
Pause Button Motif Enrichment
1.40
1.35
1.30
1.25
1.20
B
Maternal reads (M)
Paternal reads (P)
Maternal sites
Paternal sites
1.0
0.1
10
-10
0
10
20
Distance from pause site (bp)
C
Initiator Motif Enrichment
1.70
1.65
1.60
1
2
3
4
log(PI)
D
0.42
0.86
0.92
Initiator Motif Enrichment
1.80
1.75
1.70
1.65
1.60
1.55
NELF Subunits:
None
NELF−B and C/D
All, or lacking NELF−E

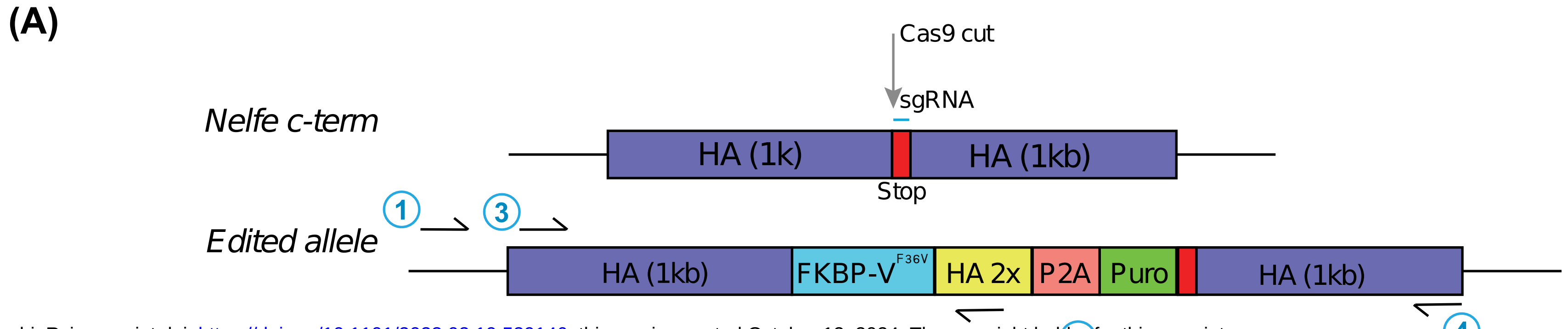

bioRxiv preprint doi: https://doi.org/10.1101/2023.02.19.529146; this version posted October 12, 2024. The copyright holder for this preprint (which was not certified by peer review) is the author/funder, who has granted bioRxiv a license to display the preprint in perpetuity. It is made available under aCC-BY 4.0 International license.

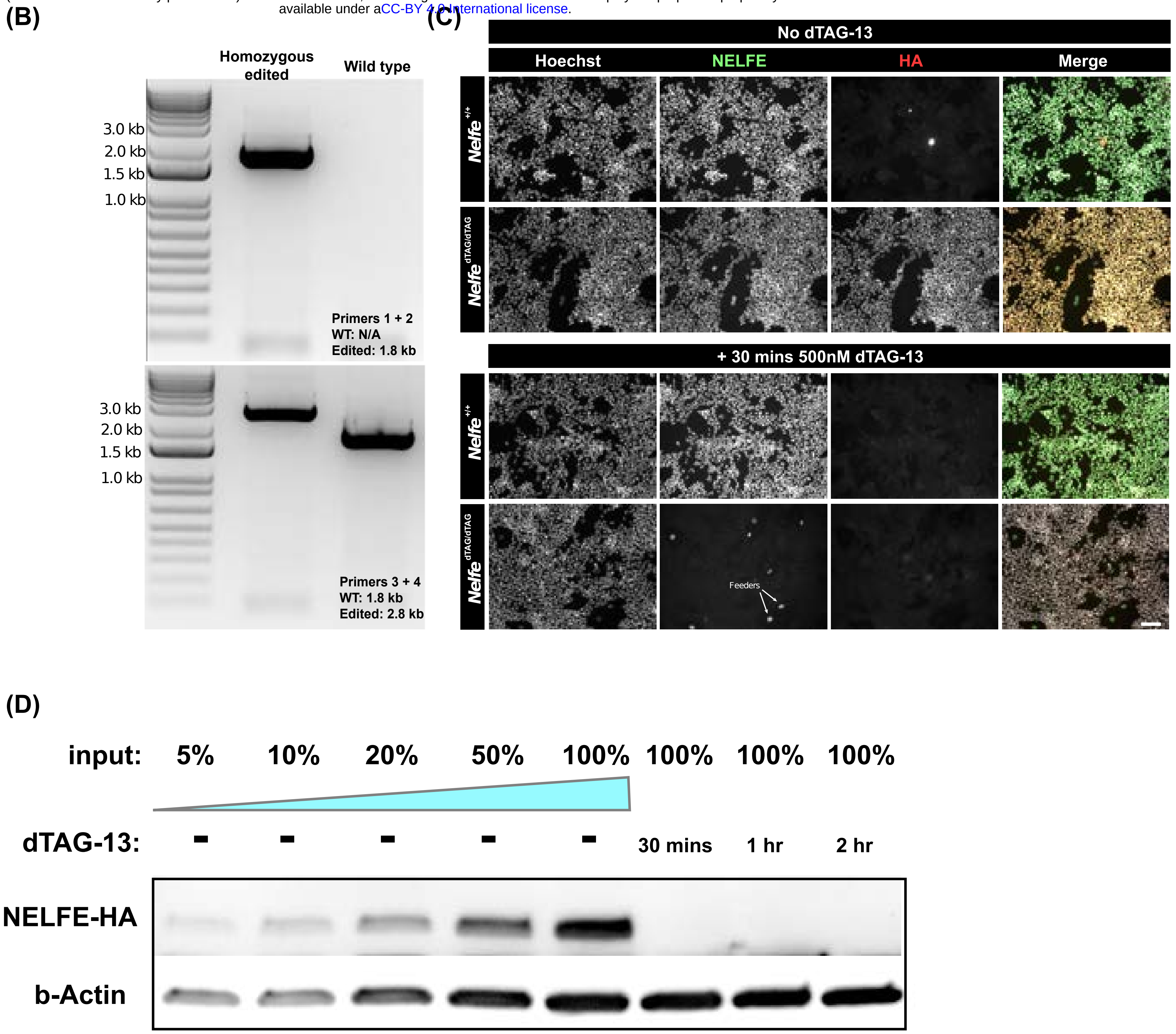

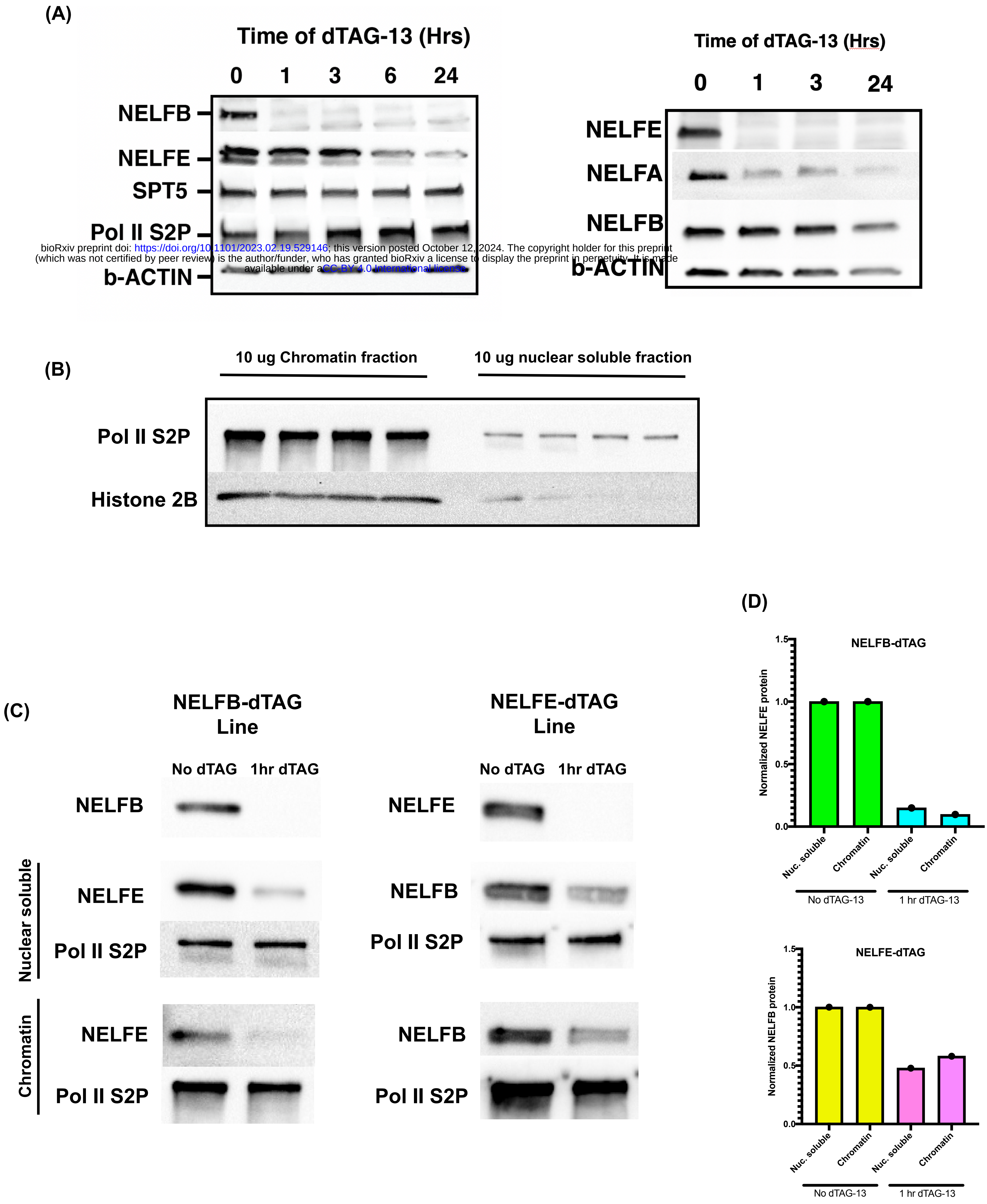

bioRxiv preprint doi: https://doi.org/10.1101/2023.02.19.529146; this version posted October 12, 2024. The copyright holder for this preprint (which was not certified by peer review) is the author/funder, who has granted bioRxiv a license to display the preprint in perpetuity. It is made available under aCC-BY 4.0 International license.

bioRxiv preprint doi: https://doi.org/10.1101/2023.02.19.529146; this version posted October 12, 2024. The copyright holder for this preprint (which was not certified by peer review) is the author/funder, who has granted bioRxiv a license to display the preprint in perpetuity. It is made available under aCC-BY 4.0 International license.

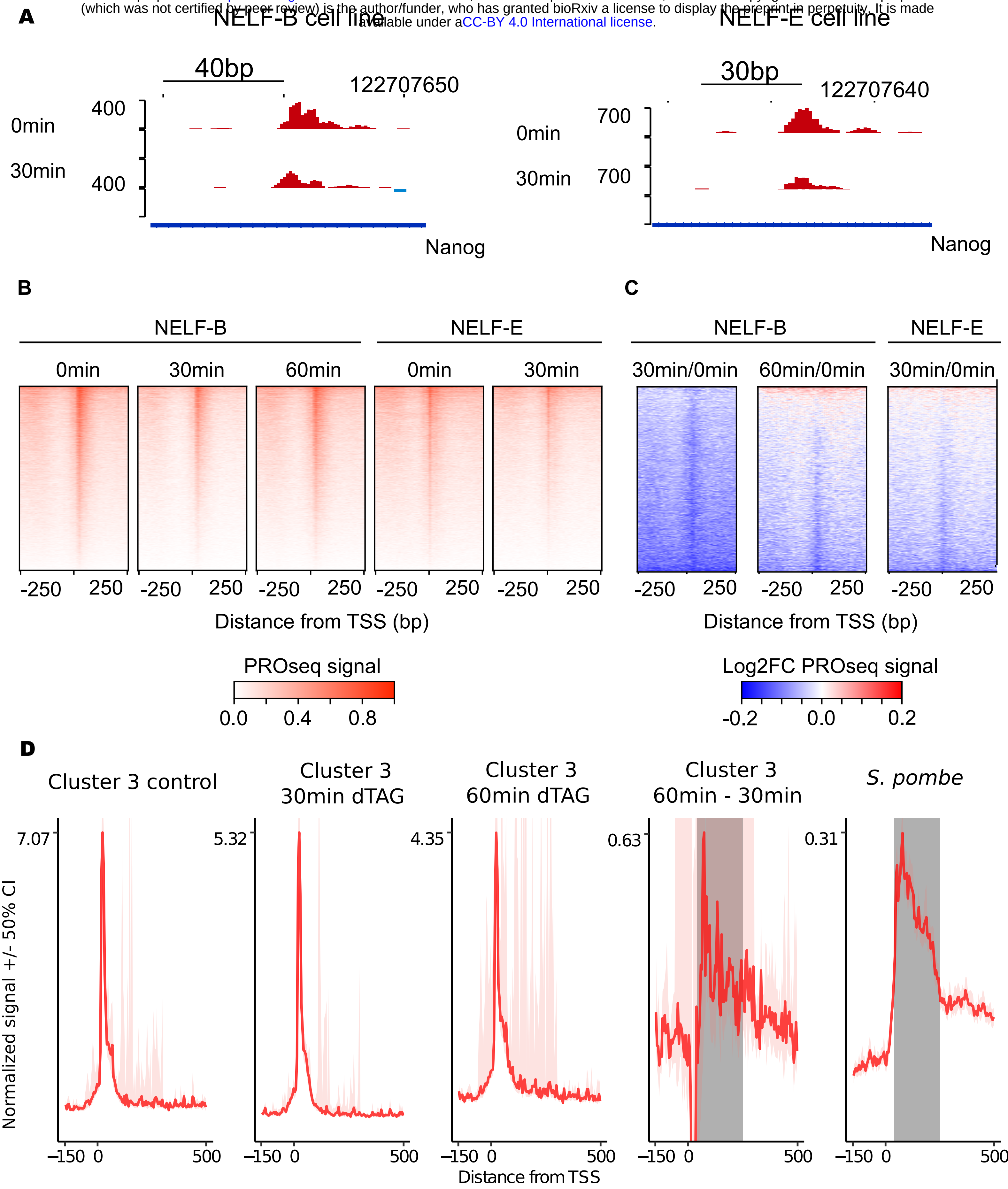

bioRxiv preprint doi: https://doi.org/10.1101/2023.02.19.529146; this version posted October 12, 2024. The copyright holder for this preprint (which was not certified by peer review) is the author/funder, who has granted bioRxiv a license to display the preprint in perpetuity. It is made available under aCC-BY 4.0 International license.

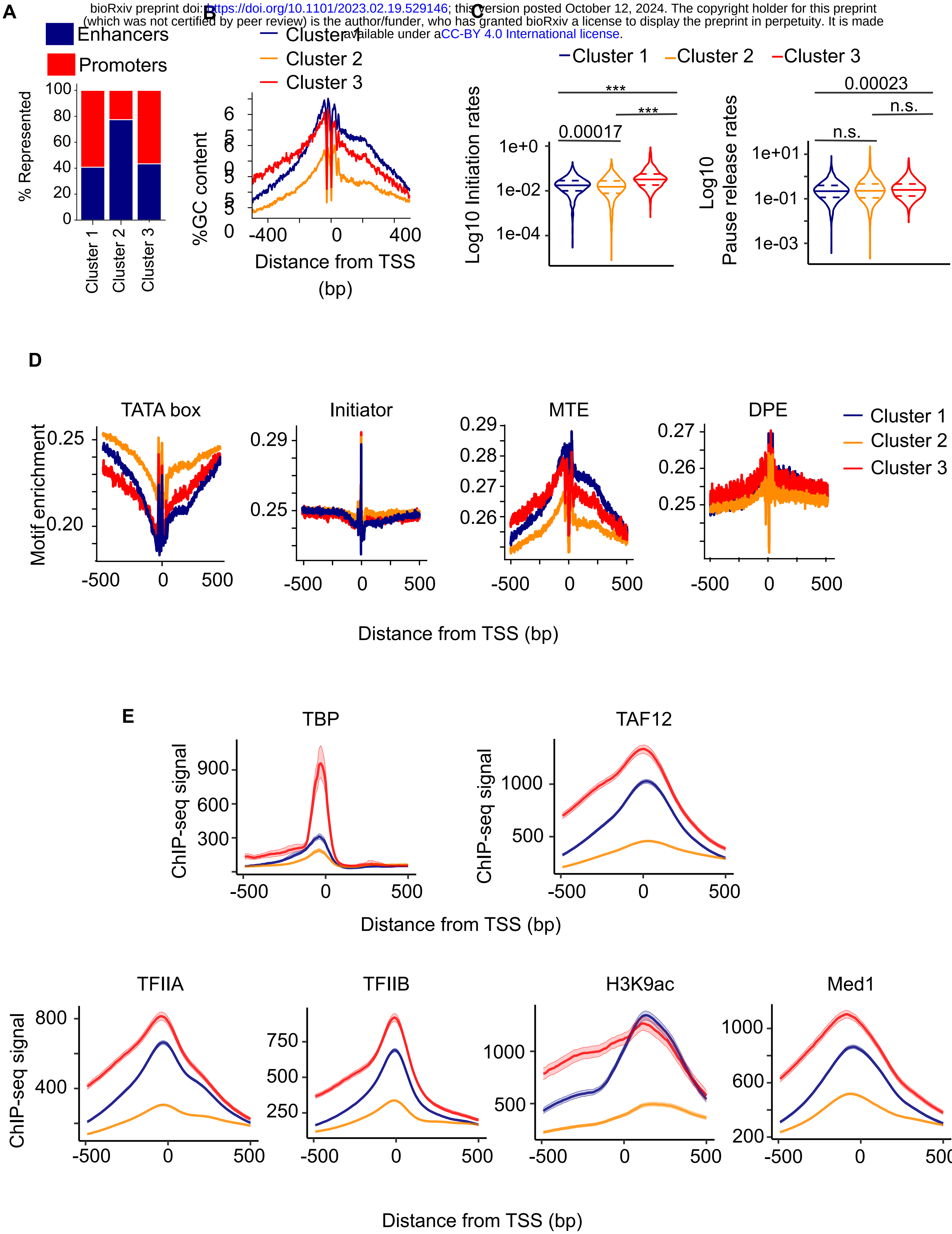

bioRxiv preprint doi: https://doi.org/10.1101/2023.02.19.529146; this version posted October 12, 2024. The copyright holder for this preprint (which was not certified by peer review) is the author/funder, who has granted bioRxiv a license to display the preprint in perpetuity. It is made available under aCC-BY 4.0 International license.

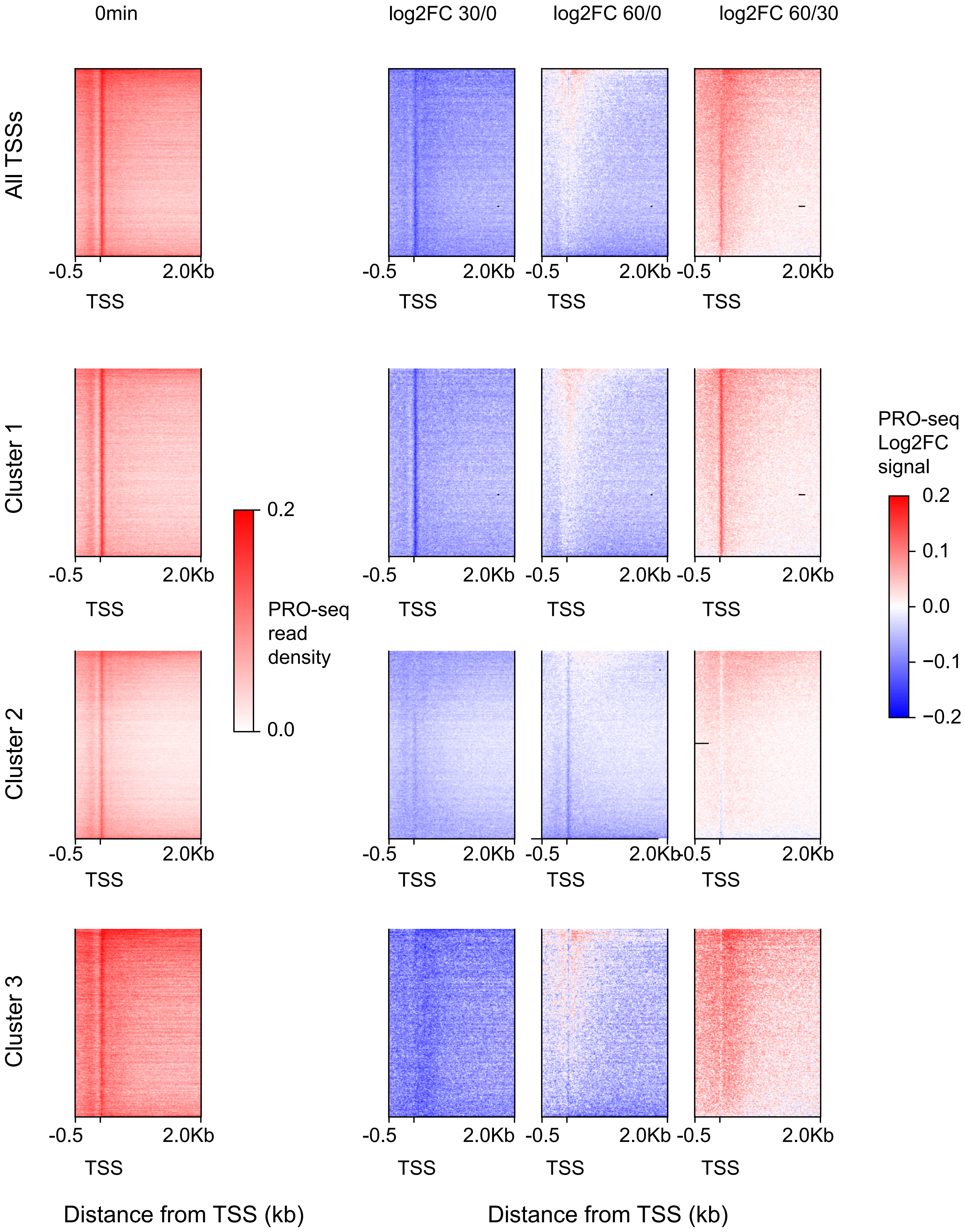

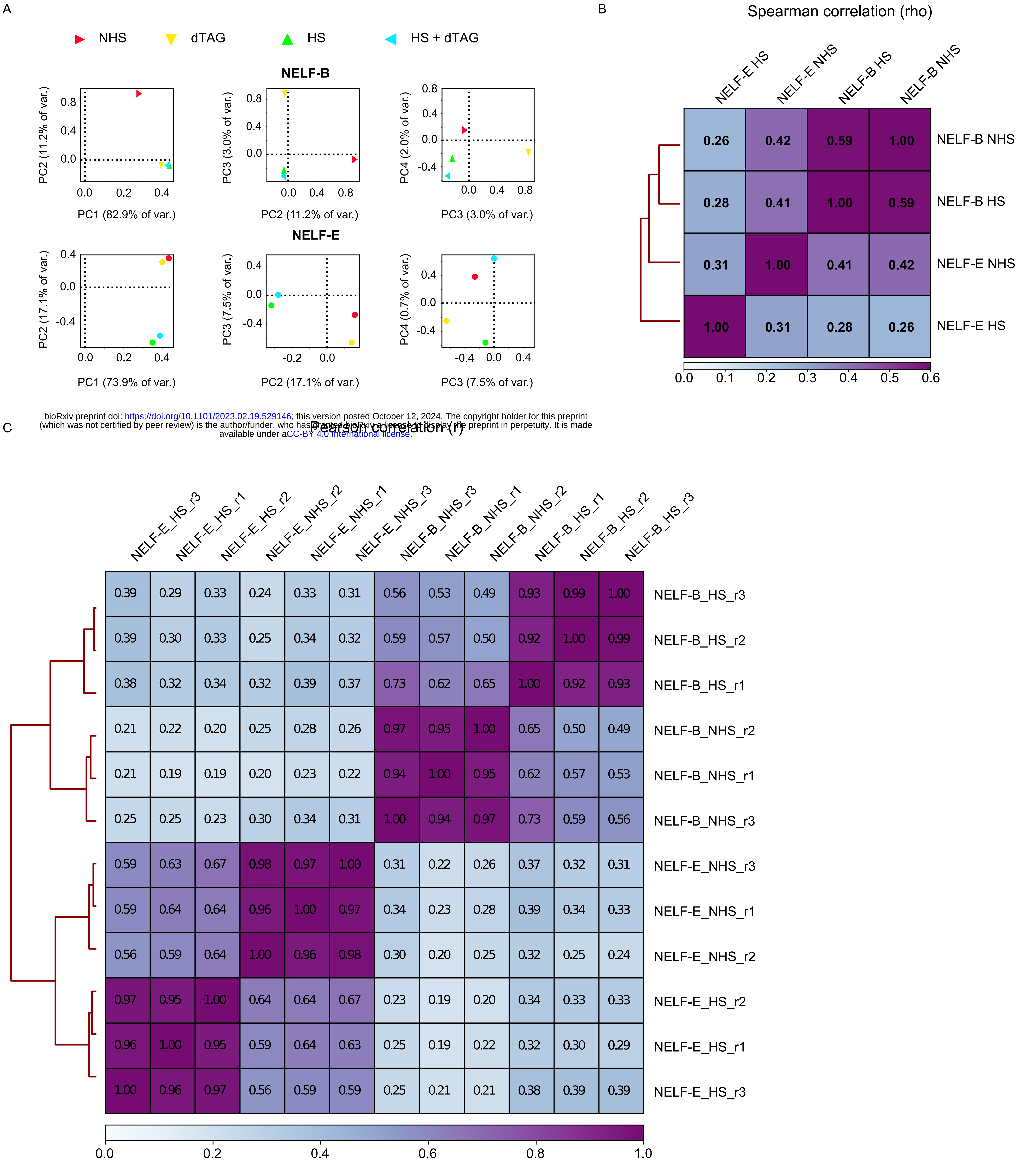

bioRxiv preprint doi: https://doi.org/10.1101/2023.02.19.529146; this version posted October 12, 2024. The copyright holder for this preprint (which was not certified by peer review) is the author/funder, who has granted bioRxiv a license to display the preprint in perpetuity. It is made available under aCC-BY 4.0 International license.

bioRxiv preprint doi: https://doi.org/10.1101/2023.02.19.529146; this version posted October 12, 2024. The copyright holder for this preprint (which was not certified by peer review) is the author/funder, who has granted bioRxiv a license to display the preprint in perpetuity. It is made available under aCC-BY 4.0 International license.

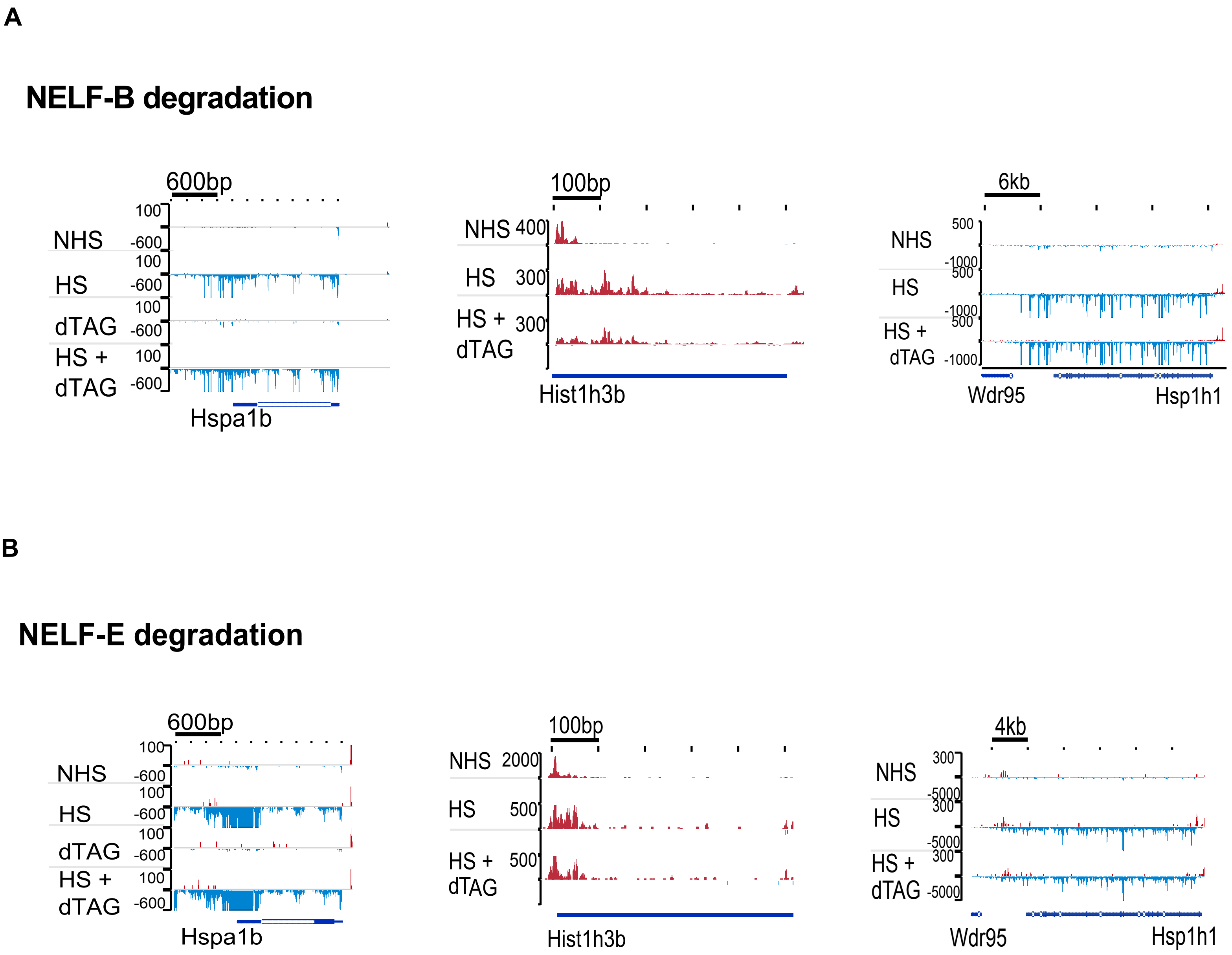

bioRxiv preprint doi: https://doi.org/10.1101/2023.02.19.529146; this version posted October 12, 2024. The copyright holder for this preprint (which was not certified by peer review) is the author/funder, who has granted bioRxiv a license to display the preprint in perpetuity. It is made available under aCC-BY 4.0 International license.

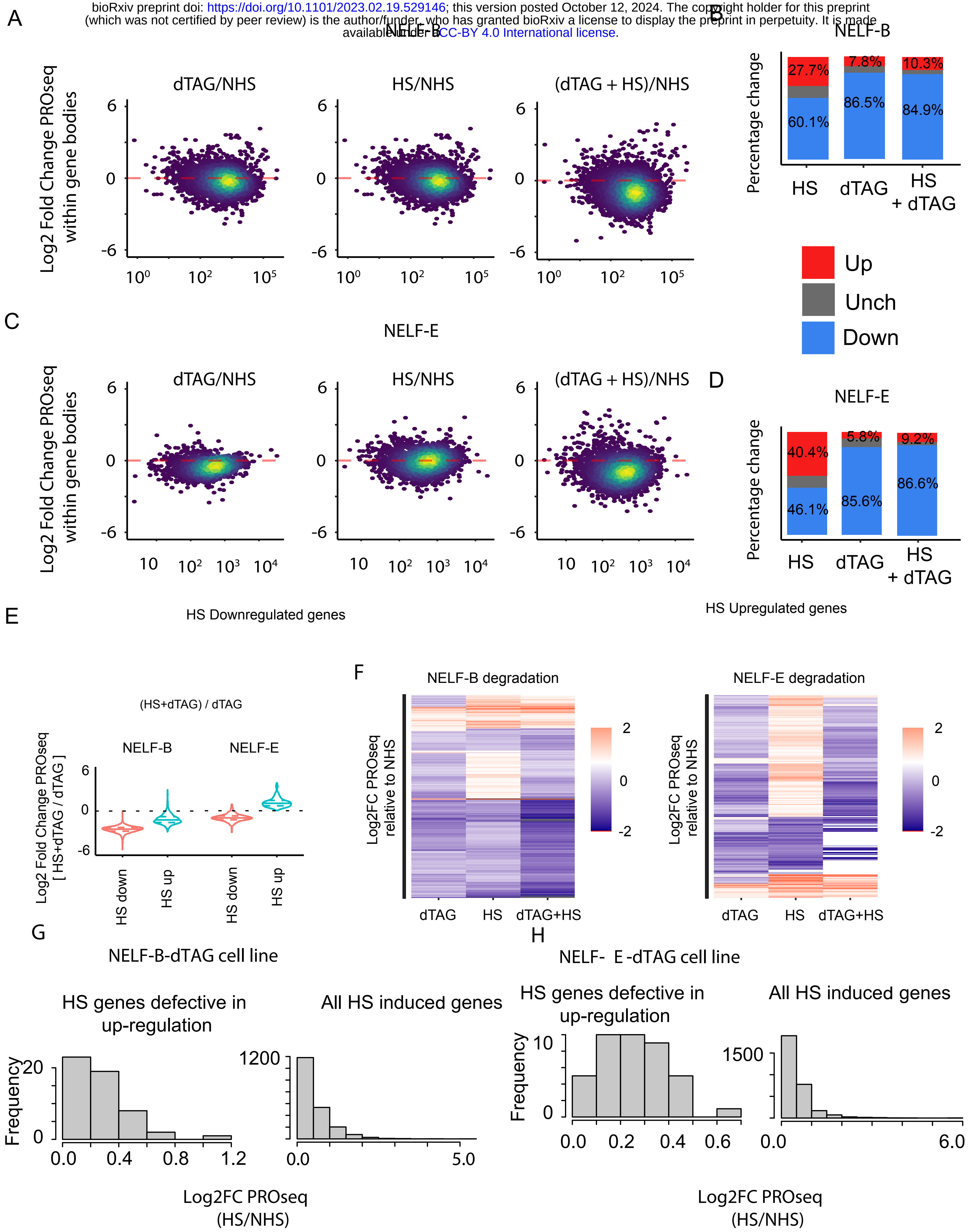

Supplement: Supplement 2 [file NIHPP2023.02.19.529146v2-supplement-2.pdf]
